# Analysis of METTL3 and METTL14 in hepatocellular carcinoma

**DOI:** 10.18632/aging.103959

**Published:** 2020-11-06

**Authors:** Xiangxiang Liu, Jian Qin, Tianyi Gao, Chenmeng Li, Xiaoxiang Chen, Kaixuan Zeng, Mu Xu, Bangshun He, Bei Pan, Xueni Xu, Yuqin Pan, Huiling Sun, Tao Xu, Shukui Wang

**Affiliations:** 1 School of Medicine, Southeast University, Nanjing 210096, Jiangsu, China; 2 General Clinical Research Center, Nanjing First Hospital, Nanjing Medical University, Nanjing 210006, Jiangsu, China; 3 Jiangsu Collaborative Innovation Center on Cancer Personalized Medicine, Nanjing Medical University, Nanjing 211100, Jiangsu, China

**Keywords:** METTL3, METTL14, N6-methyladenosine, hepatocellular carcinoma, bioinformatics analysis

## Abstract

N6-methyladenosine (m6A) RNA methylation is the most prevalent modification of messenger RNAs (mRNAs) and catalyzed by a multicomponent methyltransferase complex (MTC), among which methyltransferase-like 3 (METTL3) and METTL14 are two core molecules. However, METTL3 and METTL14 play opposite regulatory roles in hepatocellular carcinoma (HCC). Based on The Cancer Genome Atlas (TCGA) database and Gene Expression Omnibus (GEO) database, we conducted a multi-omics analysis of METTL3 and METTL14 in HCC, including RNA-sequencing, m6ARIP-sequencing, and ribosome-sequencing profiles. We found that the expression and prognostic value of METTL3 and METTL14 are opposite in HCC. Besides, after METTL3 and METTL14 knockdown, most of the dysregulated mRNAs, signaling pathways and biological processes are distinct in HCC, which partly explains the contrary regulatory role of METTL3 and METTL14. Intriguingly, these mRNAs whose stability or translation efficiency are influenced by METTL3 or METTL14 in an m6A dependent manner, jointly regulate multiple signaling pathways and biological processes, which supports the cooperative role of METTL3 and METTL14 in catalyzing m6A modification. In conclusion, our study further clarified the contradictory role of METTL3 and METTL14 in HCC.

## INTRODUCTION

N6-methyladenosine (m6A) RNA methylation, the most prevalent modification of messenger RNAs (mRNAs), accounts for almost half of the total methylated ribonucleotides [1]. In general, m6A modification is present in the transcripts of over 7,000 genes in mammalian cells, and it prefers to occur at the consensus RRACH motif (R = G or A; H = A, C, or U). Transcriptome-wide m6A site mapping reveals more details on its localization which preferentially enriches at coding sequence (CDS), around stop codon, and 3’untranslated region (3’UTR) in the transcriptomes [2, 3]. The formation of m6A is catalyzed by a multicomponent methyltransferase complex (MTC), among which methyltransferase-like 3 (METTL3), METTL14, and Wilms’ tumor 1-associating protein (WTAP), Vir Like M6A Methyltransferase Associated (KIAA1429), RNA Binding Motif Protein 15 (RBM15), and Zinc Finger CCCH-Type Containing 13 (ZC3H13) have been detected [4–8]. The m6A is a reversible modification that can also be removed by RNA demethylases, including fat mass and obesity-associated protein (FTO) and alkylated DNA repair protein alkB homolog 5 (ALKBH5) [9, 10]. In addition, m6A modified mRNAs can be bound by multiple specific RNA binding proteins, of which the known ones are YTH Domain-Containing Protein 1 (YTHDC1), YTH Domain Family, Member 1/2/3 (YTHDF1/2/3), Insulin-Like Growth Factor 2 MRNA Binding Protein 1/2/3 (IGF2BP1/2/3) [11–15].

METTL3 and METTL14, two core components of MTC, colocalize in nuclear speckles, and catalyze the covalent transfer of a methyl group to adenine in a heterodimer form [16]. The unusual m6A modification caused by differentially expressed METTL13 or METTL14 plays a critical role in the malignant progression of various cancers, such as bladder cancer, gastric cancer, and hepatocellular carcinoma (HCC) [17–19]. Interestingly, emerging evidence indicated an opposite regulatory role of METTL3 and METTL14 in several cancers, such as glioblastoma [20, 21], HCC [19, 22], and colorectal cancer (CRC) [23, 24]. METTL3 was demonstrated to be upregulated in CRC and to facilitate CRC progression by maintaining the SRY-Box 2 (SOX2) mRNA stability [23]. However, our recent study proved that METTL14 is significantly downregulated in CRC and suppresses cell growth and metastasis via regulating primary miR-375 processing [25]. Consistently, METTL3 serves as an oncogene, but METTL14 is a tumor suppressor in HCC [19, 22]. Study *in vitro* showed that both METTL3 and METTL14 have a methyltransferase domain to methylate RNA and the m6A methyltransferase activity is much higher in METTL3/METTL14 complex than that in either subunit alone [4]. However, the crystal structure and biochemical evidence suggested that METTL3, rather than METTL14, is the unique catalytic subunit, and METTL14 functions in structural stabilization and RNA substrate recognition [26]. Therefore, it is urgently needed to further clarify the characterization of METTL3 and METTL14 in cancers.

In this study, based on The Cancer Genome Atlas (TCGA) database and Gene Expression Omnibus (GEO) database, we conducted a multi-omics analysis of METTL3 and METTL14 in HCC. We validated the reverse expression and prognostic value of METTL3 and METTL14 in HCC and discovered that most of the mRNAs and associated signaling pathways and biological processes regulated by METTL3 and METTL14 are different, which may be partly responsible for their contrary functions in HCC. However, these mRNAs whose stability or translation efficiency (TE) are affected by METTL3 or METTL14 in an m6A dependent manner, jointly regulate multiple signaling pathways and biological processes, such as TGF-beta signaling pathway, protein ubiquitination, and cell cycle.

## RESULTS

### The opposite expression and prognostic value of METTL3 and METTL14 in HCC

**Figure 1:**
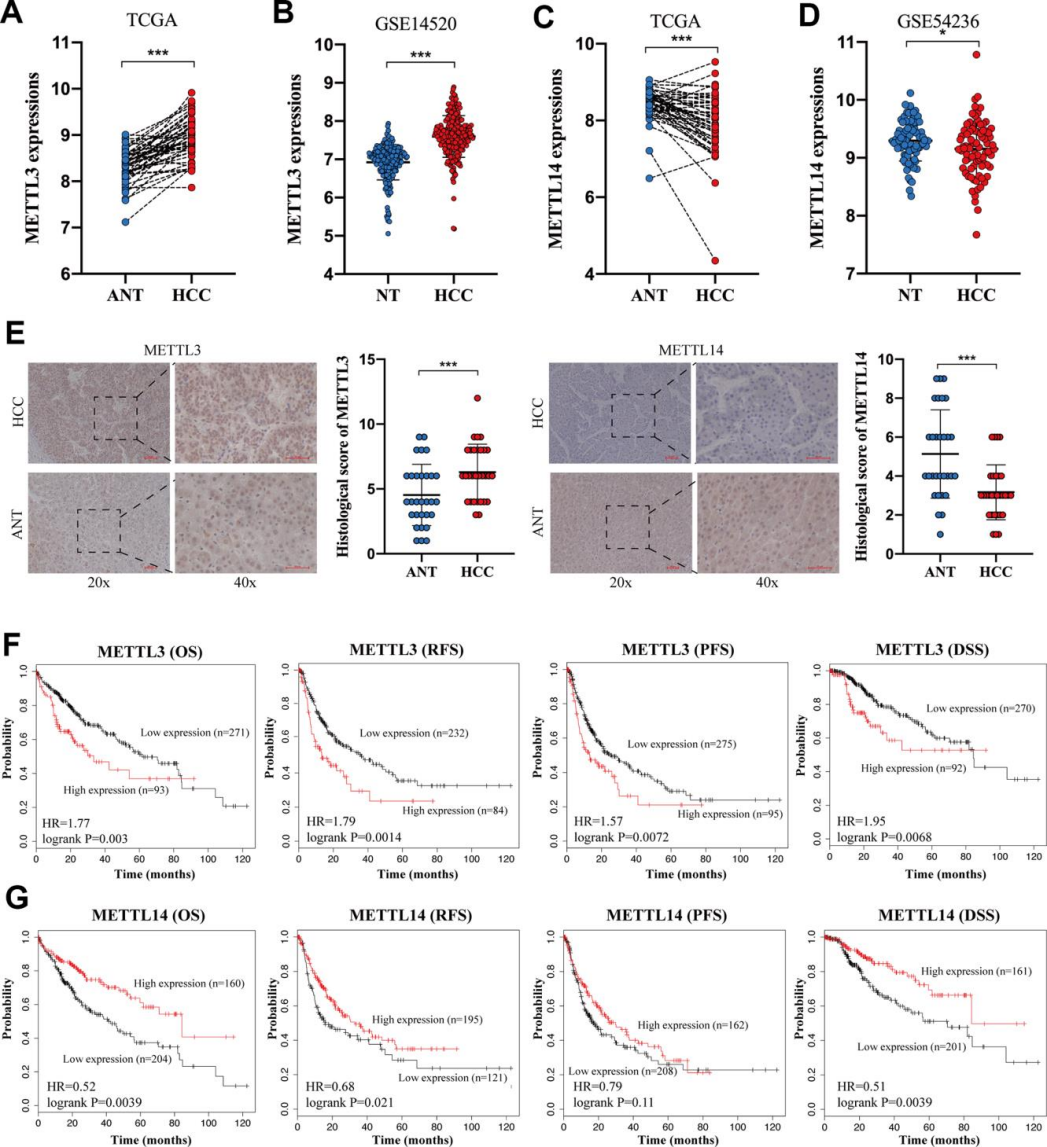
**The opposite expression and prognostic value of METTL3 and METTL14 in HCC.** (**A**, **B**) The expression of METTL3 in HCC tissues and NTs based on the TCGA database and GSE14520 analysis. (**C**, **D**) The expression of METTL14 in HCC tissues and NTs based on the TCGA database and GSE14520 analysis. (**E**) IHC analysis of METTL3 and METTL14 in HCC tissues and adjacent NTs. (**F**) The associations between METTL3 expression and OS, RFS, PFS, and DSS of HCC patients. (**G**) The associations between METTL14 expression and OS, RFS, PFS, and DSS of HCC patients. * p<0.05, *** p<0.001.

To verify the expression of METTL3 and METTL14 in HCC, we analyzed the TCGA database and two GEO datasets. TCGA database and GSE14520 [27] analysis showed an increased expression of METTL3 in HCC tissues compared to normal liver tissues (NTs) (Figure 1A, 1B). In contrast, The TCGA database and GSE54236 [28] analysis exhibited a downregulated expression of METTL14 in HCC tissues (Figure 1C and 1D). IHC analysis validated the overexpressed METTL3 and downregulated METTL14 expression in HCC (Figure 1E). Besides, we discovered that HCC patients with higher METTL3 expression have shorter overall survival (OS) time, relapse-free survival (RFS) time, progression-free survival (PFS) time, and disease-specific survival (DSS) time compared to those with low METTL3 expression (Figure 1F). Conversely, HCC patients with low METTL14 expression undergo poorer OS rate, RFS rate, PFS rate, and DSS rate compared to those with high METTL14 expression (Figure 1G). These results demonstrated an opposite expression and prognostic value of METTL3 and METTL14 in HCC.

### These differentially expressed genes regulated by METTL3 and METTL14 knockdown participate in different signaling pathways and biological processes

**Figure 2:**
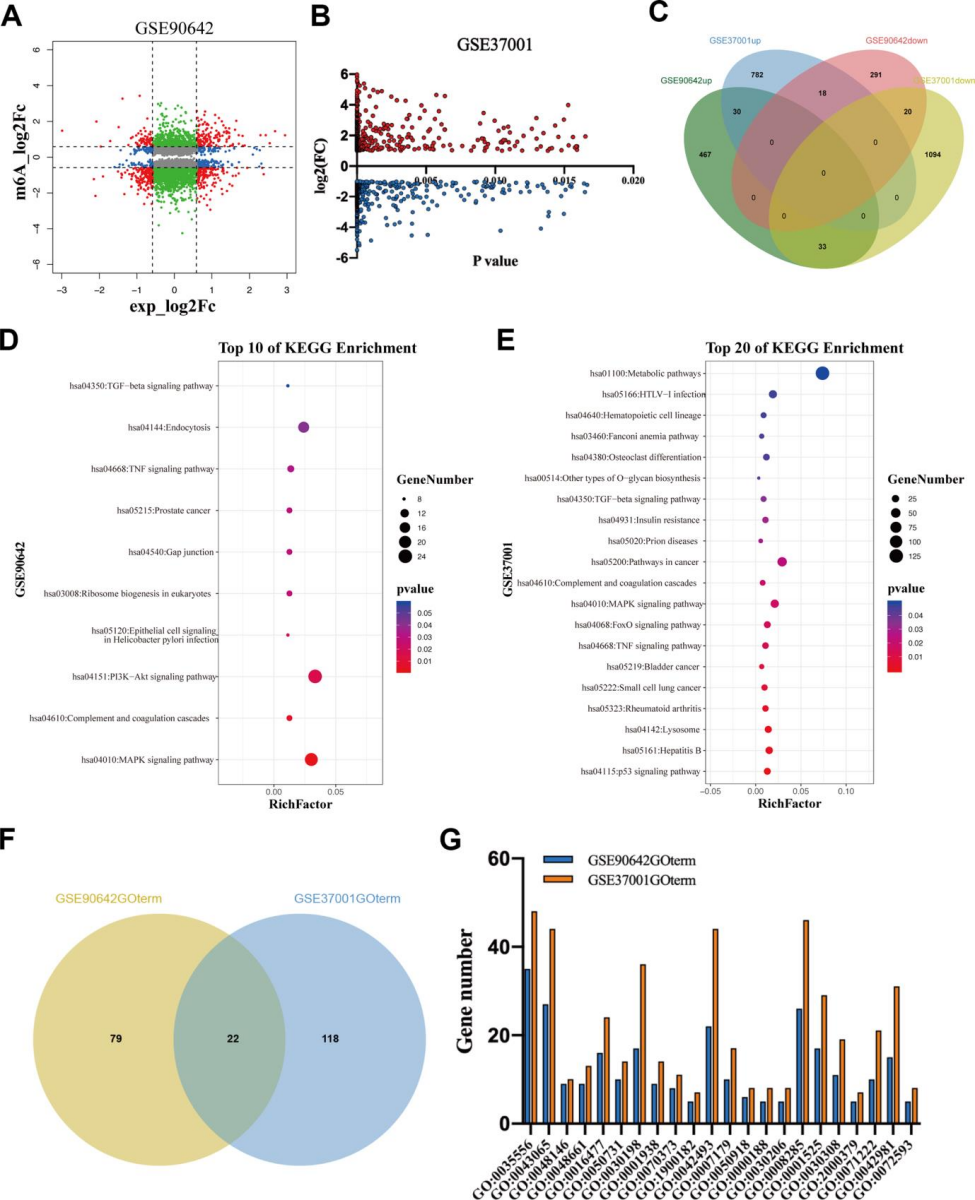
**The DEGs regulated by METTL3 and METTL14 knockdown participate in different signaling pathways and biological processes.** (**A**, **B**) The DEGs regulated by METTL3 and METTL14 knockdown. (**C**) Integrated analysis of M3DEGs and M14DEGs. (**D**, **E**) KEGG pathway analysis of M3DEGs and M14DEGs. (**F**) The number of biological processes regulated by M3DEGs and M14DEGs. (**G**) The common biological processes regulated by M3DEGs and M14DEGs.

To explore the regulatory role of METTL3 and METTL14 on mRNA expression, we analyzed GSE90642 and GSE37001 datasets [2, 15] The RNA sequencing data of GSE90642 showed that 329 and 530 mRNAs were downregulated and overexpressed in HepG2 cells after METTL14 knockdown, respectively (Figure 2A). In addition, METTL14 knockdown resulted in 705 mRNAs with downregulated m6A modification. Interestingly, 270 mRNAs with upregulated m6A modification were also observed (Figure 2A). GSE37001 analysis revealed that 1147 downregulated mRNAs and 812 overexpressed mRNAs after METTL3 knockdown in HepG2 cells (Figure 2A). Intriguingly, when overlapping these differentially expressed genes (DEGs), we found that only a small number of DEGs (n=101) were co-regulated by METTL3 and METTL14, of which 51% even showed an opposite expression (Figure 2C). Thereafter, we conducted a KEGG analysis of these DEGs. Kyoto Encyclopedia of Genes and Genomes (KEGG) analysis revealed that the DEGs regulated by METTL14 (M14DEGs) were significantly enriched in ten signaling pathways, such as MAPK, PI3K-Akt, and TGF-beta signaling pathways (Figure 2D). Meanwhile, these DEGs regulated by METTL3 (M3DEGs) were significantly enriched in twenty signaling pathways, such as p53, TNF, and TGF-beta signaling pathways (Figure 2E). As expected, only three signaling pathways were jointly regulated by METTL3 and METTL14, including MAPK, TNF, and TGF-beta signaling pathways. Subsequently, we carried out Gene Ontology (GO) analysis of M14DEGs and M3DEGs, respectively (Additional file 1: Supplementary Table 1, 2). In line with KEGG analysis results, only a small part of biological processes (n=22) was jointly regulated by METTL3 and METTL14, such as cell migration (GO:0016477) (Figure 2F, 2G). Taken together, we concluded that most M3DEGs and M14DEGs as well as associated signaling pathways and biological processes are different in HCC.

### The signaling pathways and biological processes of m6A modified M3DEGs

**Figure 3:**
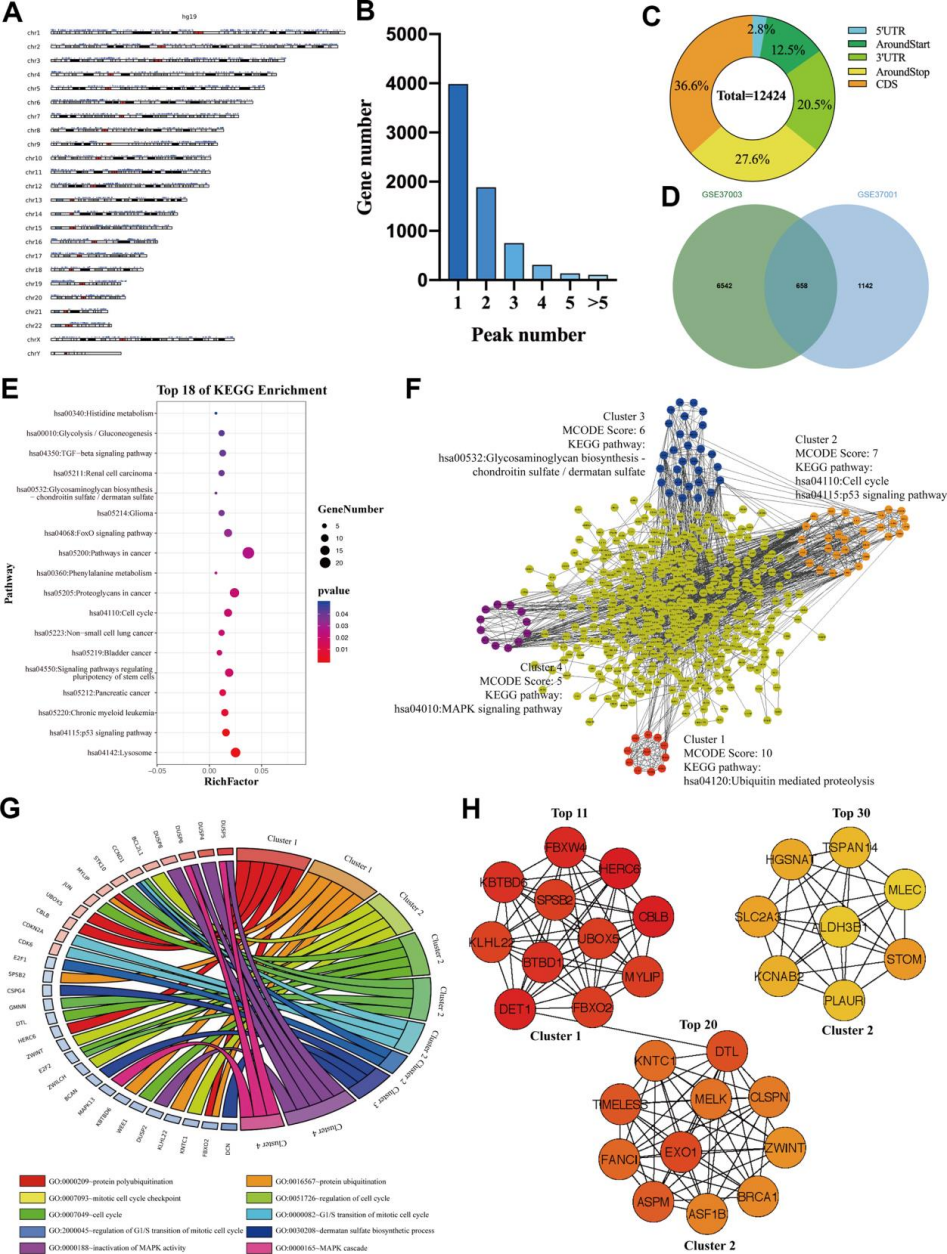
**The signaling pathways and biological processes of m6A modified M3DEGs.** (**A**) The chromosome location of transcripts having m6A peaks in HepG2 cell. (**B**) The number of genes with various m6A peaks. (**C**) The distribution of m6A peaks in transcripts. (**D**) Integrated analysis of M3DEGs and mRNAs with m6A peaks. (**E**) KEGG pathway analysis of m6A modified M3DEGs. (**F**, **G**) KEGG pathway and GO enrichment analysis of functional molecular clusters among the PPI network of m6A modified M3DEGs. (**H**) The top 30 hub genes among m6A modified M3DEGs.

To investigate whether those M3DEGs were m6A modified genes, we first analyzed m6ARIP-sequencing data in HepG2 cells by using GSE37003 [2] which revealed a total of 12424 m6A peaks in 7180 transcripts. Chromosome location analysis showed that these m6A peaks were enriched in all chromosomes (Figure 3A). Besides, most transcripts (82%) had only one or two m6A peaks (Figure 3B). Consistent with previous reports, most m6A peaks (85%) located in CDS, around stop codon, and 3' UTR of mRNAs (Figure 3C). Unexpectedly, when we overlapped m6A modified transcripts with M3DEGs, we found that only 37% of M3DEGs had m6A peaks (Figure 3D). KEGG pathway analysis showed that these m6A modified M3DEGs were enriched in 18 signaling pathways, such as p53 signaling pathway, signaling pathways regulating pluripotency of stem cells, and TGF-beta signaling pathway (Figure 3E). After that, we conducted a protein-protein interaction (PPI) network of these m6A modified M3DEGs which detected four functional molecular clusters (Figure 3F). KEGG pathway analysis of these clusters exhibited three common signaling pathways, including cell cycle and p53 signaling pathway of cluster 2, Glycosaminoglycan biosynthesis-chondroitin sulfate/dermatan sulfate of cluster 3. In addition, Cluster 1 and cluster 4 revealed two unique pathways, including Ubiquitin mediated proteolysis and the MAPK signaling pathway (Figure 3F). Consistent with KEGG pathway analysis, GO enrichment analysis showed that cluster 1 was involved in protein polyubiquitination and ubiquitination, that cluster 2 was involved in the cell cycle, that cluster 3 was involved in dermatan sulfate biosynthetic process, and that cluster 4 was involved in MAPK activity (Figure 3G). Moreover, all top 30 hub genes among m6A modified M3DEGs belonged to cluster 1 and cluster 2 (Figure 3H). Meanwhile, the expression of most hub genes was positively correlated with METTL3 expression in HCC (87%) and significantly associated with the OS of HCC patients (73%) (Additional file 2: Supplementary Table 3, 4). Based on the above data, we believed that METTL3 directly regulates a small number of genes’ expression in an m6A dependent manner and that these m6A modified M3DEGs are mainly involved in protein ubiquitination and cell cycle.

### The signaling pathways and biological processes of m6A modified M14DEGs

**Figure 4:**
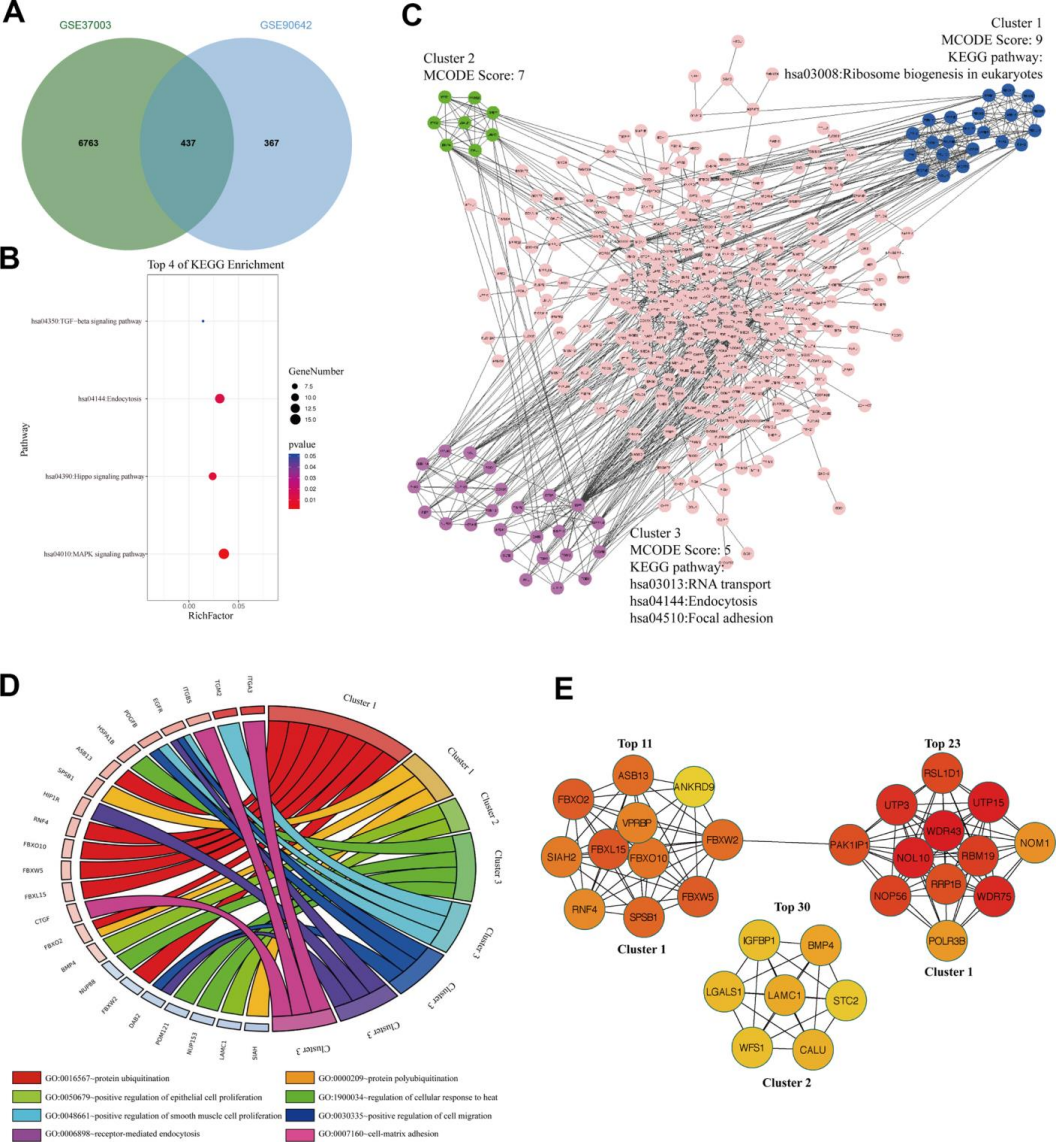
**The signaling pathways and biological processes of m6A modified M14DEGs.** (**A**) Integrated analysis of M14DEGs and mRNAs with m6A peaks. (**B**) KEGG pathway analysis of m6A modified M14DEGs. (**C**, **D**) KEGG pathway and GO enrichment analysis of functional molecular clusters among the PPI network of m6A modified M14DEGs. (**E**) The top 30 hub genes among m6A modified M14DEGs.

To analyze the m6A modified M14DEGs, we overlapped m6A modified genes with M14DEGs. We found that 54% of M14DEGs were m6A modified mRNAs (Figure 4A). KEGG pathway analysis showed that these m6A modified M14DEGs were enriched in only 4 signaling pathways, including the MAPK signaling pathway, Hippo signaling pathway, Endocytosis, and TGF-beta signaling pathway (Figure 4B). PPI network of these m6A modified M14DEGs revealed three functional molecular clusters (Figure 4C). KEGG pathway analysis of cluster 3 showed a common signaling pathway, Endocytosis. Interestingly, cluster 1 and cluster 3 revealed three unique pathways, including Ribosome biogenesis in eukaryotes, RNA transport, and Focal adhesion (Figure 4C). GO enrichment analysis showed that cluster 1 was associated with protein polyubiquitination and ubiquitination, and that cluster 2 was associated with regulation of epithelial cell proliferation, and that cluster 3 was associated with cell proliferation, migration, and adhesion (Figure 4D). Furthermore, all top 30 hub genes among m6A modified M14DEGs belonged to cluster 1 and cluster 2 (Figure 4E). Moreover, consistently, the expression of most hub genes was positively correlated with METTL14 expression in HCC (73%) and significantly associated with the OS of HCC patients (50%) (Additional file 3: Supplementary Table 5, 6). Overall, our results indicated that METTL14, liking METTL3, directly regulates part of genes’ expression in an m6A dependent manner and that these m6A modified M14DEGs mainly participate in protein ubiquitination and cell proliferation.

### These genes with changed TE regulated by METTL3 and METTL14 knockdown participate in distinct signaling pathways and biological processes

**Figure 5:**
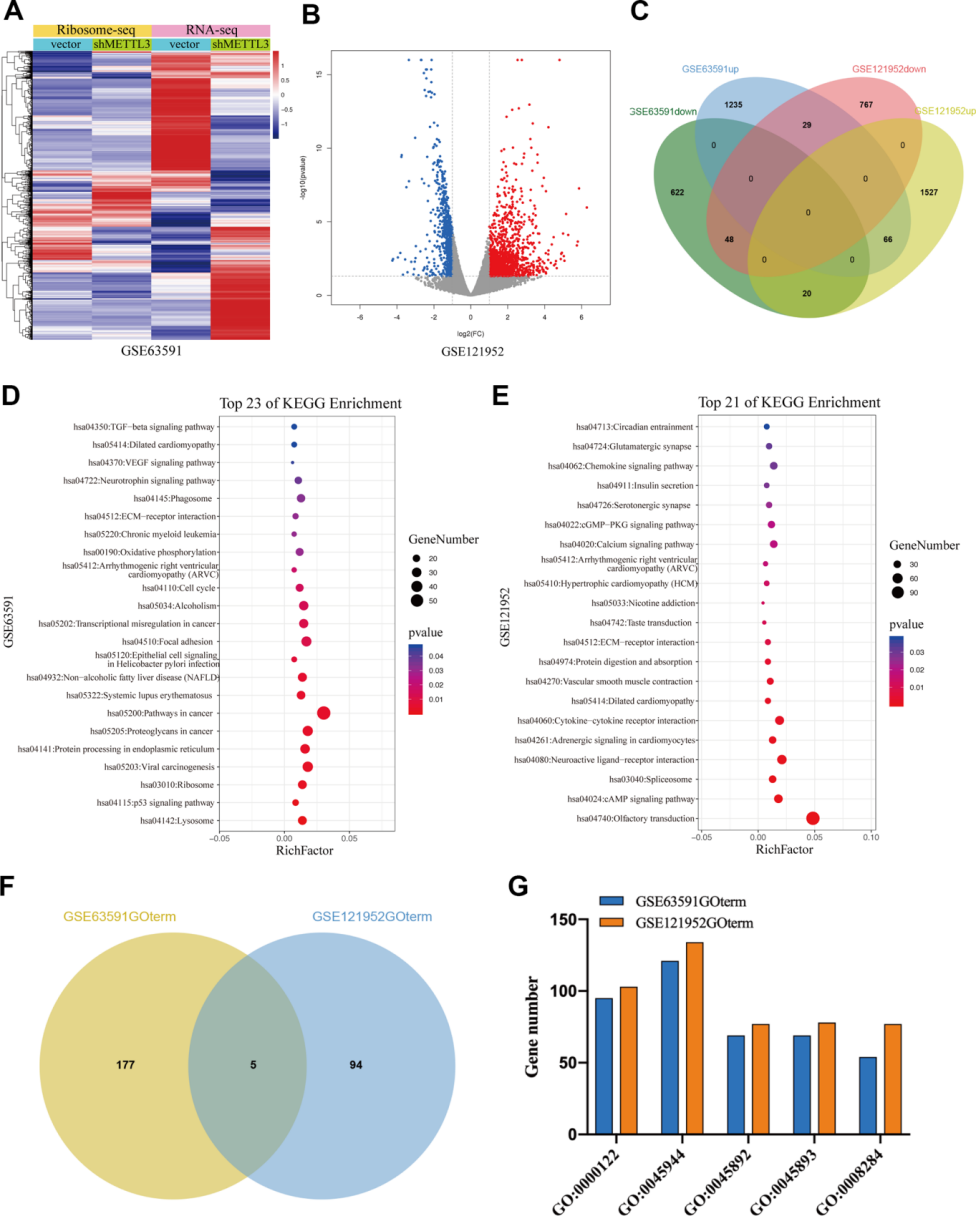
**The TEGs regulated by METTL3 and METTL14 knockdown participate in distinct signaling pathways and biological processes.** (**A**, **B**) The TEGs regulated by METTL3 and METTL14 knockdown. (**C**) Integrated analysis of M3TEGs and M14TEGs. (**D**, **E**) KEGG pathway analysis of M3TEGs and M14TEGs. (**F**) The number of biological processes regulated by M3TEGs and M14TEGs. (**G**) The common biological processes regulated by M3TEGs and M14TEGs.

To further investigate the regulatory role of METTL3 and METTL14 on mRNA translation, we analyzed GSE63591 [12] and GSE121952 [29] datasets. Ribosome sequencing data of GSE63591 showed that the TE of 690 mRNAs was significantly downregulated while the TE of 1330 mRNAs was significantly upregulated after METTL3 knockdown (Figure 5A). GSE121952 analysis exhibited that after METTL14 knockdown, the TE of 844 and 1613 mRNAs was significantly downregulated and upregulated, respectively (Figure 5B). As expected, when we overlapped these TEGs, we found that only a small part (n=163) was regulated by METTL3 and METTL14 collectively (Figure 5C). As shown in Figure 5D, KEGG pathway analysis showed that these TEGs regulated by METTL3 (M3TEGs) were significantly enriched in 23 signaling pathways, such as Lysosome, cell cycle, and p53 signaling pathway, and that these TEGs regulated by METTL14 (M14TEGs) were significantly enriched in 21 distinct signaling pathways, such as cAMP signaling pathway, Spliceosome, and Protein digestion and absorption (Figure 5E). Subsequently, GO enrichment analysis of these TEGs was analyzed (Additional file 4: Supplementary Table 7, 8), which demonstrated that only a small part of biological processes (n=5) was regulated by METTL3 and METTL14 corporately, including regulation of transcription (GO:0000122, GO:0045944, GO:0045892, and GO:0045893) and cell proliferation (GO:0008284) (Figure 5F, 5G). Together, we proved that most of M3TEGs and M14TEGs are different and involved with distinct signaling pathways and biological processes in HCC.

### The signaling pathways and biological processes of m6A modified M3TEGs

**Figure 6:**
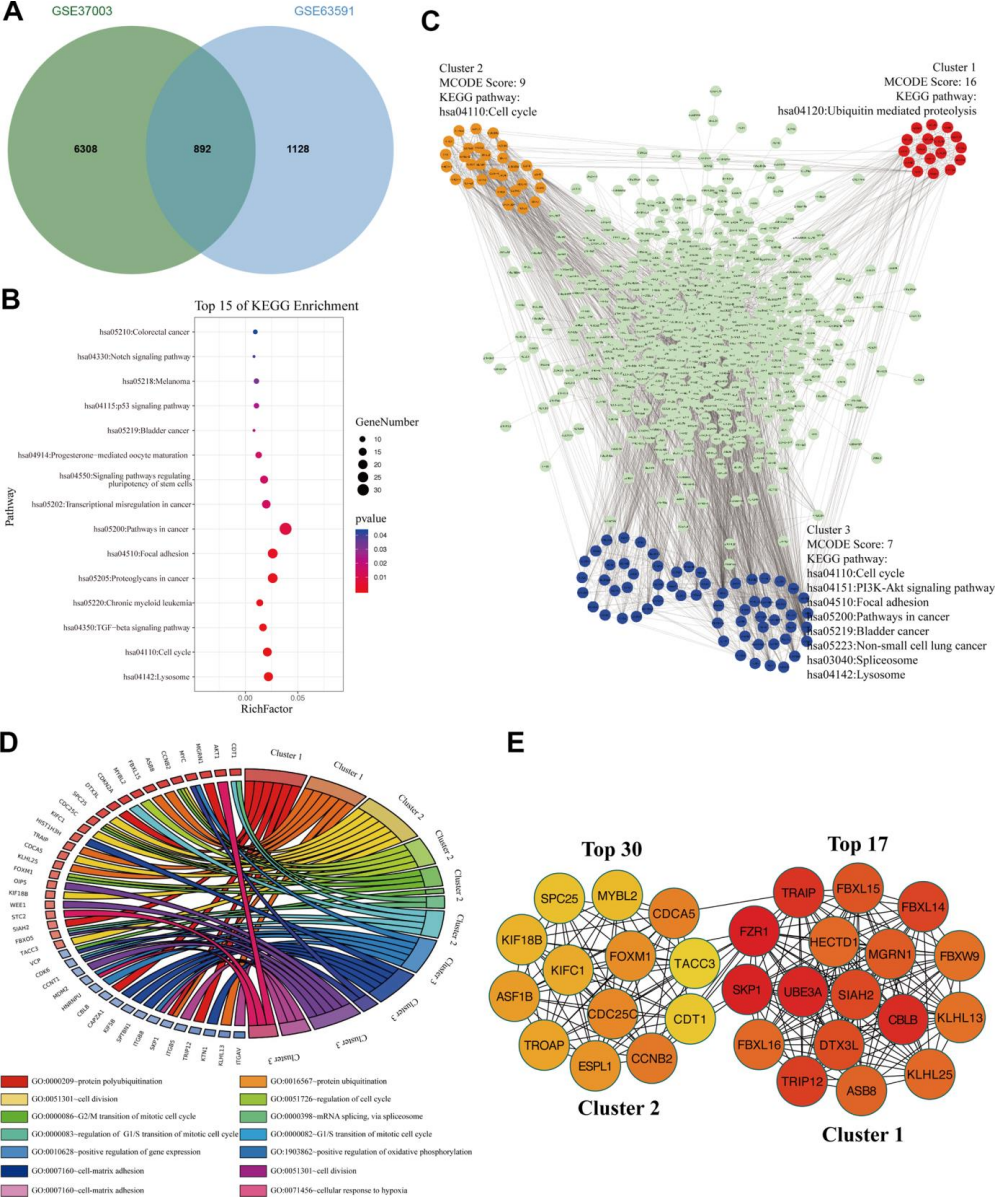
**The signaling pathways and biological processes of m6A modified M3TEGs.** (**A**) Integrated analysis of M3TEGs and mRNAs with m6A peaks. (**B**) KEGG pathway analysis of m6A modified M3TEGs. (**C**, **D**) KEGG pathway and GO enrichment analysis of functional molecular clusters among PPI network of m6A modified M3TEGs. (**E**) The top 30 hub genes among m6A modified M3TEGs.

To analyze these M3TEGs with m6A modification, we overlapped m6A modified transcripts with M3TEGs, which showed that 44% of M3TEGs were directly regulated by m6A modification (Figure 6A). KEGG pathway analysis showed that these m6A modified M3TEGs were significantly associated with 15 signaling pathways, including Lysosome, Cell cycle, TGF-beta signaling pathway, Chronic myeloid leukemia, Proteoglycans in cancer, Focal adhesion, Pathways in cancer, Transcription misregulation in cancer, Signaling pathways regulating pluripotency of stem cells, Progesterone-mediated oocyte maturation, Bladder cancer, p53 signaling pathway, Melanoma, Notch signaling pathway, and Colorectal cancer (Figure 6B). PPI network of these m6A modified M3TEGs revealed three functional molecular clusters (Figure 6C). KEGG pathway analysis of cluster 2 and cluster 3 exhibited five common signaling pathways, including Cell cycle, Lysosome, Bladder cancer, Pathways in cancer, and Focal adhesion. Besides, cluster 1 and cluster 3 revealed several unique pathways, including Ubiquitin mediated proteolysis, PI3K-Akt signaling pathway, Non-small cell lung cancer, and Spliceosome (Figure 6C). GO analysis showed that cluster 1 regulated protein polyubiquitination and ubiquitination, and that cluster 2 regulated cell cycle, and that cluster 3 regulated cell division and adhesion (Figure 6D). Furthermore, all top 30 hub genes among m6A modified M3TEGs were enriched in cluster 1 and cluster 2 (Figure 6E). In addition, the expression of most hub genes was positively correlated with METTL3 expression in HCC (97%) and significantly associated with the OS of HCC patients (67%) (Additional file 5: Supplementary Table 9, 10). The data described above suggested that METTL3 directly regulates part of mRNAs TE through catalyzing m6A modification, and that like m6A modified M3DEGs, these m6A modified M3TEGs are also mainly involved with protein ubiquitination and cell cycle.

### The signaling pathways and biological processes of m6A modified M14TEGs

**Figure 7:**
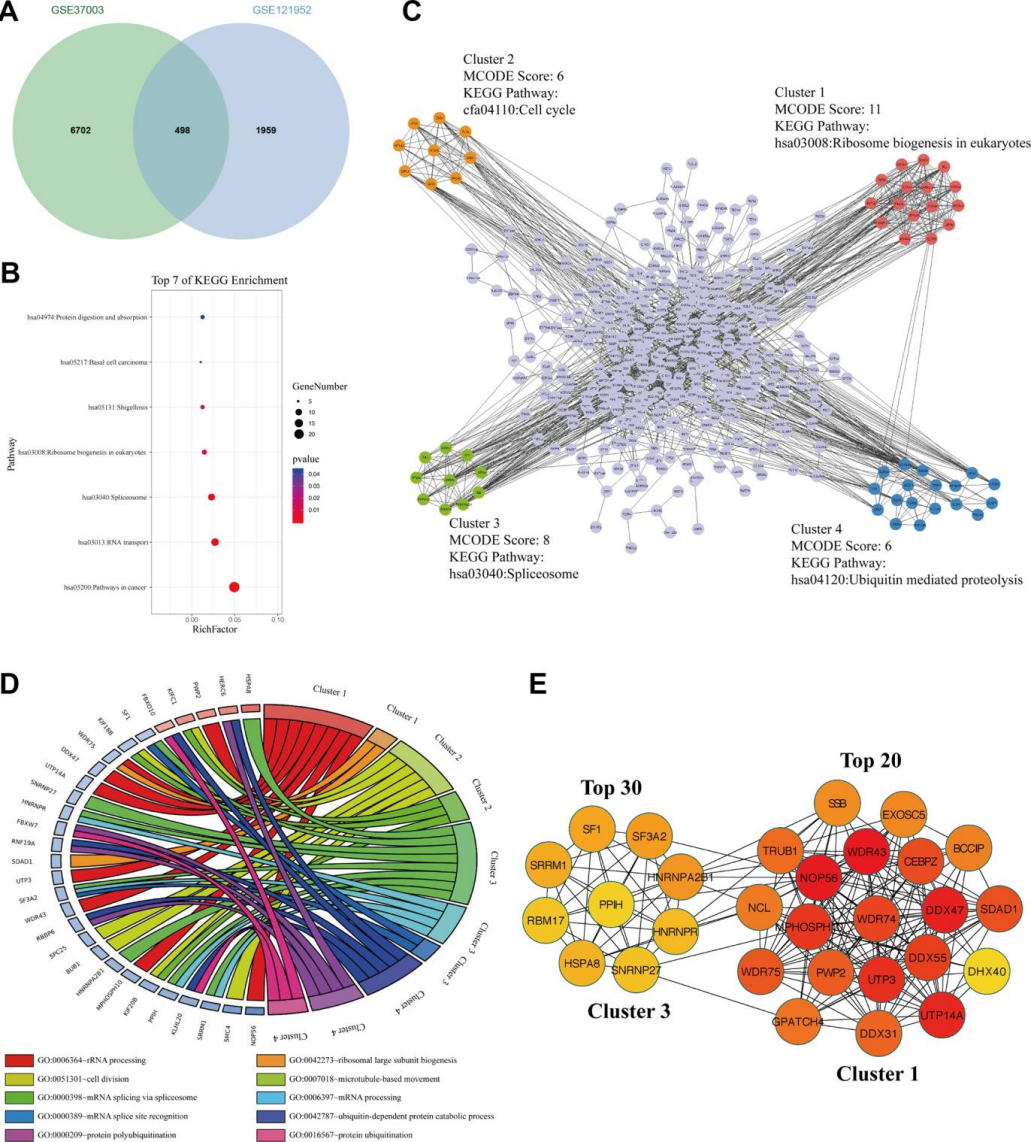
**The signaling pathways and biological processes of m6A modified M14TEGs.** (**A**) Integrated analysis of M14TEGs and mRNAs with m6A peaks. (**B**) KEGG pathway analysis of m6A modified M14TEGs. (**C**, **D**) KEGG pathway and GO enrichment analysis of functional molecular clusters among the PPI network of m6A modified M14TEGs. (**E**) The top 30 hub genes among m6A modified M14TEGs.

To explore the m6A modified M14TEGs, we overlapped m6A modified genes with M14TEGs, which showed that only 20% of M14TEGs were m6A modified (Figure 7A). KEGG pathway analysis exhibited that these m6A modified M14TEGs were significantly enriched in 7 signaling pathways, including pathways in cancer, RNA transport, Spliceosome, Ribosome biogenesis in eukaryotes, Shigellosis, Basal cell carcinoma, and Protein digestion and absorption (Figure 7B). PPI network of these m6A modified M14TEGs revealed four functional molecular clusters (Figure 7C). KEGG pathway analysis of cluster 1 and cluster 3 showed two common signaling pathways, including Ribosome biogenesis in eukaryotes and Spliceosome. Additionally, cluster 2 and cluster 4 added two unique pathways, including Cell cycle and Ubiquitin mediated proteolysis (Figure 7C). GO enrichment analysis showed that cluster 1 participated in rRNA processing and ribosomal large subunit biogenesis, and that cluster 2 participated in cell division and microtubule-based movement, and that cluster 3 participated in mRNA splice and processing, and that cluster 4 participated in ubiquitin-dependent protein catabolic process, protein polyubiquitination and ubiquitination (Figure 7D). Moreover, all top 30 hub genes among m6A modified M14TEGs were enriched in cluster 1 and cluster 3 (Figure 7E). Meanwhile, the expression of most hub genes was positively correlated with METTL3 expression in HCC (90%) and associated with the OS of HCC patients (60%) (Additional file 6: Supplementary Table 11, 12). All in all, our data indicated that METTL14 directly regulates a small number of mRNAs TE through catalyzing m6A modification and that m6A modified M14TEGs not only are associated with ribosome biogenesis and mRNA splice but also regulate cell cycle and protein ubiquitination in collaboration with M3TEGs.

## DISCUSSION

Despite chemical modifications of DNA and histones, epigenetic regulation also contains hundreds of distinct post-transcriptional modifications in cellular RNAs among which m6A is the most abundant one occurring in eukaryotic mRNAs [30, 31]. Through its direct binding with m6A binding proteins, m6A influences almost every step in the process of an mRNA molecule, from splicing, stability, and export to translation [13, 14, 32, 33]. As core molecules of MTC, METTL3 and METTL14 often perform an opposite effect on tumor process in same cancer [19, 22]. The crystal structure analysis revealed that only METTL3, but not METTL14, carries out the catalytic role and that METTL14 mainly enhances the activity of METTL3 in part through stabilizing structure [26]. In addition, METTL3 was reported to promote the translation of important oncogenes in human cancers independent of its catalytic activity and m6A binding proteins [34]. Moreover, a cancer-associated mutation in METTL14 not only decreased methyltransferase activity but also decreased the substrate specificity of the MTC, such that both the consensus sequence GGACU and the non-consensus sequence GGAUU were methylated at similar efficiencies [4]. Hence, we hypothesized that apart from the synergistic effect of METTL3 and METTL14 on catalyzing m6A modification, each molecule has its unique functions.

In this study, we found that although METTL3 or METTL14 knockdown resulted in the abnormal expression of hundreds of mRNAs in HepG2 cells, only a small part of DEGs was affected by METTL3 and METTL14 together, and even half of the expression of DEGs had the opposite change. In agreement, the analysis of the KEGG pathway and GO enrichment showed that METTL3 and METTL14 jointly regulated few signal pathways and biological processes. The above data suggested that in HCC, most of the targets and biological functions of METTL3 and METTL14 are different. Further analysis revealed that only 37% of M3DEGs and 54% of M14DEGs have m6A peaks. KEGG pathway analysis showed that these m6A modified DEGs only participated in half of the signaling pathways regulated by METTL3 and METTL14, in which the TGF-beta signaling pathway was the same one. The TGF-beta signaling pathway is an important oncogenic pathway in cancers. Our previous study demonstrated that TGF-beta promotes the metastasis of triple-negative breast cancer [35]. It is worth noting that both hub M3DEGs and M14DEGs modified by m6A were involved in protein ubiquitination. In fact, protein ubiquitination is closely related to the TGF-beta signaling pathway. For instance, the four and a half LIM-only protein 2 (FHL2) activates TGF-beta signaling by regulating the ubiquitination of the E3 ligase [36]. Our results implied that M3DEGs and M14DEGs partake in several signaling pathways and biological processes collectively in an m6A dependent manner.

Moreover, we further analyzed the effect of METTL3 and METTL14 on mRNA translation. In accordance with transcriptome analysis, METTL3 or METTL14 downregulation resulted in hundreds of TEGs. However, only a few TEGs were jointly controlled by METTL3 and METTL14. Surprisingly, KEGG pathway analysis revealed that M3TEGs and M14TEGs did not share any signaling pathway. Consistently, GO enrichment analysis showed that even fewer biological processes were co-regulated by METTL3 and METTL14 through influencing mRNA TE. These data indicated that M3TEGs and M14TEGs participate in distinct signaling pathways and biological processes. Furthermore, we found that 44% of M3TEGs and 20% of M14TEGs were m6A modified. Interestingly, most signaling pathways of M3TEGs were the same as that of M3DEGs, such as the p53 signaling pathway and cell cycle. In addition, like M3DEGs, M3TEGs also had significant enrichment in protein ubiquitination and cell cycle. An uncontrolled cell cycle is a critical stimulus to tumor progression. For example, the integration of genomic and transcriptional features in pancreatic cancer reveals an increased cell cycle progression in metastasis [37]. Indeed, protein ubiquitination is also significantly associated with cell cycle transition [38, 39]. Hence, we hypothesized that METTL3 may regulate specific mRNAs related to protein ubiquitination and cell cycle at the transcription and translation levels dependent on m6A modification, to promote the progress of HCC. Unlike M3TEGs, M14TEGs were mainly involved in mRNA splicing, and part of M14TEGs and M14DEGs co-regulated ribosome biogenesis in eukaryotes in HCC. Intriguingly, regulation of protein ubiquitination was also an important biological process of M14TEGs. Based on our results, we speculated that m6A modification regulated by METTL3 and METTL14 might be closely related to anomalous protein ubiquitination in HCC. Besides, when analyzing the hub genes in M3DEGs, M14DEGs, M3TEGs, and M14TEGs, we found that expression of most hub genes was positively correlated with METTL3 expression or METTL14 expression and served as unfavorable predictors of HCC patients’ survival.

M6A modification is a comprehensive and context-dependent biological process [40]. Transcripts with m6A modification occur in different destinies if they bind to various m6A binding proteins. For instance, IGF2BPs stabilize, but YTHDF2 degrades m6A modified mRNAs [13, 32]. Besides, miRNAs can regulate m6A abundance by modulating METTL3 binding to mRNAs [41]. Intriguingly, METTL3 and METTL14 also modulate N6-methyladenosine-dependent primary miRNA processing [19, 42]. Moreover, miRNAs can also regulate mRNAs expression through direct binding to the 3'UTRs of targets by inducing mRNAs degradation and/or translational repression [43]. Therefore, it is limited to study the role of METTL3 and METTL14 in cancer only from the perspective of catalyzing m6A modification.

There are still some deficiencies in our study. First, to identified m6A modified mRNAs, we just overlapped dysregulated mRNAs with those having m6A peaks. Whether these dysregulated mRNAs are m6A modified ones should be validated through m6ARIP-PCR. Second, the cell line used in the GSE63591 dataset is HeLa cells. Although m6A is a conserved modification [4], similar investigations in HCC cells ought to be done to verify our results. Third, our analysis only described the role of METTL3 and METTL14 in HCC, whether this hypothesis applies to other types of cancer, such as colorectal cancer and glioma, needs further investigations. Forth, the expression of hub genes, especially these in M3TEGs and M14TEGs, should be validated through IHC analysis.

In conclusion, our study further clarified the characteristics of METTL3 and METTL14 in HCC which is beneficial to further research of METTL3 and METTL14.

## MATERIALS AND METHODS

### Study cohort

The METTL3 and METTL14 expression data in NTs and HCC tissues were retrieved from the TCGA database (https://cancergenome.nih.gov/) and the GEO database (https://www.ncbi.nlm.nih.gov/geo/) (GSE14520 and GSE54236). TCGA database of HCC contains 50 matched NTs and HCC tissues. The GSE14520 dataset includes 214 NTs and 225 HCC tissues, while GSE54236 consists of 80 NTs and 81 HCC tissues. The RNA sequencing data of METTL3 knockdown and METTL14 knockdown in HepG2 cells were enrolled from GSE37001 and GSE90642, both METTL3 knockdown and METTL14 knockdown resulted from small interfering RNA (siRNA) transfection. The m6A peaks enriched in transcripts in HepG2 cells were analyzed and deposited in GSE37003. In addition, ribosome sequencing profiles about the effect of METTL3 knockdown and METTL14 knockdown on mRNA TE was analyzed by using GSE63591 and GSE121952. siRNA and short hairpin RNA (shRNA) was used to decrease METTL3 and METTL14 expression, respectively.

### Immunohistochemistry

Immunohistochemistry analysis for METTL3 and METTL14 was performed on paraffin sections using a primary antibody against METTL3 (1:2000, Proteintech, 15073-1-AP), METTL14 (1:1500, Proteintech, 26158-1-AP), and a horseradish peroxidase conjugated IgG (1:500; Invitrogen). Three high power fields (400×magnification) were randomly selected from 30 paired HCC tissues and ANTs. For histological scoring, the degree of positivity was initially classified according to scoring both the proportion of positive staining tumor cells and the staining intensities. Scores representing the proportion of positively stained tumor cells were graded as: 0 (<10%); 1 (11%-25%); 2 (26%-50%); 3 (51%-75%) and 4 (>75%). The intensity of staining was determined as: 0 (no staining); 1 (weak staining = light yellow); 2 (moderate staining = yellow brown); and 3 (strong staining = brown). The staining index (SI) was calculated as the product of staining intensity × percentage of positive tumor cells, resulting in scores of 0, 1, 2, 3, 4, 6, 8, 9, and 12. The reactivity degree was assessed by at least two pathologists independently. Informed consent was obtained from patients and the study was approved by the ethics committee of Nanjing First Hospital.

### Bioinformatics analysis

The differentially expressed METTL3 and METTL14 between HCC tissues and NTs were analyzed by using R software with the “limma” package (http://www.bioconductor.org/packages/release/bioc/html/limma.html). In TCGA database, GSE14520, and GSE54236, genes with |logFC| > 1 and p-value < 0.05 were regarded as the significantly dysregulated ones. The correlations between the two genes in HCC were identified by using the TCGA database. The associations between gene expression and prognosis of HCC patients were explored in the Kaplan-Meier plotter database (http://kmplot.com/analysis/) which is an online tool based on GEO, European Genome-phenome Archive (EGA), and TCGA databases with the median expression value as the cut-off value. The hazard ratio (HR) and log-rank p-value were calculated. Log-rank p < 0.05 was considered to be statistically significant. HR > 1 means that gene expression was negatively correlated with prognosis, while HR < 1 shows a positive correlation. Due to the different analysis platforms, we identified that in GSE90642, DEGs were those with |logFC| > 0.585 and p-value < 0.05, while in GSE37001, DEGs were those with |logFC| > 1 and p-value < 0.05. In GSE63591, TEGs were those with |logFC| > 2 and p-value < 0.05. However, in GSE121952, TEGs were those with |logFC| > 1 and p-value < 0.05. The DAVID website (https://david.ncifcrf.gov) was used to carry out GO enrichment and KEGG pathway analysis of putative targets of METTL3 and METTL14. PPI networks were constructed by using the Search Tool for the Retrieval of Interacting Genes (STRING) online tool (http://string.embl.de/) and further optimized in Cytoscape. The combined score higher than 0.40 was regarded as statistical significance. Moreover, the functional molecular complexes in the PPI network were identified automatedly by using Molecular Complex Detection (MCODE) app in Cytoscape, and the MOCODE score higher than 5.0 was regarded as statistical significance. In addition, the top 30 hub genes among PPI network were selected by the Cytohubba app with Maximal Clique Centrality (MCC) method.

### Statistical analysis

Data were expressed as mean ± SD (standard deviation) and performed by using GraphPad Prism 8 (GraphPad, USA) software. The difference between groups was tested by two-tail Student’s paired or unpaired t-test. p < 0.05 was considered to be statistically significant.

## Supplementary Material

Supplementary Table 1

Supplementary Table 2

Supplementary Tables 3, 4, 5 and 6

Supplementary Table 7

Supplementary Table 8

Supplementary Tables 9, 10, 11 and 12
