# Supplementary material for: Analysis of METTL3 and METTL14 in hepatocellular carcinoma
Source: Aging (Albany NY). 2020 Nov 6;12(21):21638–59. doi: 10.18632/aging.103959 (PMC7695415; doi:10.18632/aging.103959)
Supplement: Supplementary Table 1 [file aging-12-103959-s001..docx]

**Supplementary Table 1. GO analysis of DEGs regulated by METTL14.**

| **Term** | **Count** | **%** | **PValue** |
| --- | --- | --- | --- |
| GO:0098609~cell-cell adhesion | 34 | 4.40414508 | 1.27E-08 |
| GO:0035556~intracellular signal transduction | 35 | 4.53367876 | 3.58E-05 |
| GO:0045892~negative regulation of transcription, DNA-templated | 40 | 5.18134715 | 5.40E-05 |
| GO:0043065~positive regulation of apoptotic process | 27 | 3.49740933 | 1.90E-04 |
| GO:0006461~protein complex assembly | 15 | 1.94300518 | 2.20E-04 |
| GO:0046718~viral entry into host cell | 12 | 1.55440415 | 3.30E-04 |
| GO:0010951~negative regulation of endopeptidase activity | 15 | 1.94300518 | 3.42E-04 |
| GO:0048008~platelet-derived growth factor receptor signaling pathway | 7 | 0.90673575 | 8.67E-04 |
| GO:0050921~positive regulation of chemotaxis | 5 | 0.64766839 | 9.73E-04 |
| GO:0048146~positive regulation of fibroblast proliferation | 9 | 1.16580311 | 0.00128839 |
| GO:0045893~positive regulation of transcription, DNA-templated | 36 | 4.66321244 | 0.00163626 |
| GO:0032967~positive regulation of collagen biosynthetic process | 6 | 0.77720207 | 0.00186665 |
| GO:0045737~positive regulation of cyclin-dependent protein serine/threonine kinase activity | 6 | 0.77720207 | 0.00228119 |
| GO:0030330~DNA damage response, signal transduction by p53 class mediator | 5 | 0.64766839 | 0.00243774 |
| GO:0048661~positive regulation of smooth muscle cell proliferation | 9 | 1.16580311 | 0.00256879 |
| GO:0031396~regulation of protein ubiquitination | 5 | 0.64766839 | 0.00314842 |
| GO:0035025~positive regulation of Rho protein signal transduction | 6 | 0.77720207 | 0.00330488 |
| GO:0016477~cell migration | 16 | 2.07253886 | 0.00387915 |
| GO:0048568~embryonic organ development | 5 | 0.64766839 | 0.00398827 |
| GO:0032930~positive regulation of superoxide anion generation | 4 | 0.51813472 | 0.0044726 |
| GO:0070374~positive regulation of ERK1 and ERK2 cascade | 16 | 2.07253886 | 0.00455746 |
| GO:0035793~positive regulation of metanephric mesenchymal cell migration by platelet-derived growth factor receptor-beta signaling pathway | 3 | 0.38860104 | 0.00467008 |
| GO:0097421~liver regeneration | 6 | 0.77720207 | 0.0054054 |
| GO:0050731~positive regulation of peptidyl-tyrosine phosphorylation | 10 | 1.29533679 | 0.00540732 |
| GO:0030198~extracellular matrix organization | 17 | 2.20207254 | 0.00554089 |
| GO:0030111~regulation of Wnt signaling pathway | 5 | 0.64766839 | 0.00609573 |
| GO:0001938~positive regulation of endothelial cell proliferation | 9 | 1.16580311 | 0.00613718 |
| GO:0030335~positive regulation of cell migration | 16 | 2.07253886 | 0.00718963 |
| GO:1902895~positive regulation of pri-miRNA transcription from RNA polymerase II promoter | 5 | 0.64766839 | 0.00738217 |
| GO:0014911~positive regulation of smooth muscle cell migration | 5 | 0.64766839 | 0.00738217 |
| GO:0070373~negative regulation of ERK1 and ERK2 cascade | 8 | 1.03626943 | 0.00814715 |
| GO:0002576~platelet degranulation | 11 | 1.42487047 | 0.00820509 |
| GO:0000462~maturation of SSU-rRNA from tricistronic rRNA transcript (SSU-rRNA, 5.8S rRNA, LSU-rRNA) | 6 | 0.77720207 | 0.00830701 |
| GO:1900182~positive regulation of protein localization to nucleus | 5 | 0.64766839 | 0.00883536 |
| GO:0000154~rRNA modification | 3 | 0.38860104 | 0.00909251 |
| GO:0008360~regulation of cell shape | 13 | 1.68393782 | 0.01053646 |
| GO:0048024~regulation of mRNA splicing, via spliceosome | 4 | 0.51813472 | 0.01070899 |
| GO:0036499~PERK-mediated unfolded protein response | 4 | 0.51813472 | 0.01070899 |
| GO:0042493~response to drug | 22 | 2.84974093 | 0.01091576 |
| GO:0007179~transforming growth factor beta receptor signaling pathway | 10 | 1.29533679 | 0.0112943 |
| GO:0050918~positive chemotaxis | 6 | 0.77720207 | 0.01214056 |
| GO:0006364~rRNA processing | 17 | 2.20207254 | 0.01238493 |
| GO:0014066~regulation of phosphatidylinositol 3-kinase signaling | 9 | 1.16580311 | 0.01260404 |
| GO:0009611~response to wounding | 8 | 1.03626943 | 0.01264182 |
| GO:0007010~cytoskeleton organization | 14 | 1.8134715 | 0.01275822 |
| GO:0046854~phosphatidylinositol phosphorylation | 10 | 1.29533679 | 0.01289615 |
| GO:0030177~positive regulation of Wnt signaling pathway | 6 | 0.77720207 | 0.01364636 |
| GO:0007088~regulation of mitotic nuclear division | 5 | 0.64766839 | 0.01427137 |
| GO:0045648~positive regulation of erythrocyte differentiation | 5 | 0.64766839 | 0.01427137 |
| GO:0000188~inactivation of MAPK activity | 5 | 0.64766839 | 0.01646424 |
| GO:0030206~chondroitin sulfate biosynthetic process | 5 | 0.64766839 | 0.01646424 |
| GO:0030194~positive regulation of blood coagulation | 4 | 0.51813472 | 0.01669546 |
| GO:0008285~negative regulation of cell proliferation | 26 | 3.36787565 | 0.01685039 |
| GO:0030168~platelet activation | 11 | 1.42487047 | 0.01699057 |
| GO:0071333~cellular response to glucose stimulus | 7 | 0.90673575 | 0.017132 |
| GO:0001525~angiogenesis | 17 | 2.20207254 | 0.01763624 |
| GO:0051092~positive regulation of NF-kappaB transcription factor activity | 12 | 1.55440415 | 0.01784477 |
| GO:0051056~regulation of small GTPase mediated signal transduction | 12 | 1.55440415 | 0.01876545 |
| GO:0030307~positive regulation of cell growth | 9 | 1.16580311 | 0.01906074 |
| GO:1904707~positive regulation of vascular smooth muscle cell proliferation | 4 | 0.51813472 | 0.02025989 |
| GO:0045190~isotype switching | 4 | 0.51813472 | 0.02025989 |
| GO:0043280~positive regulation of cysteine-type endopeptidase activity involved in apoptotic process | 6 | 0.77720207 | 0.02091401 |
| GO:0031397~negative regulation of protein ubiquitination | 6 | 0.77720207 | 0.02305912 |
| GO:0030308~negative regulation of cell growth | 11 | 1.42487047 | 0.02337025 |
| GO:0042733~embryonic digit morphogenesis | 7 | 0.90673575 | 0.02392011 |
| GO:0051895~negative regulation of focal adhesion assembly | 4 | 0.51813472 | 0.02420869 |
| GO:0016358~dendrite development | 5 | 0.64766839 | 0.02425891 |
| GO:2000352~negative regulation of endothelial cell apoptotic process | 5 | 0.64766839 | 0.02425891 |
| GO:0022008~neurogenesis | 6 | 0.77720207 | 0.02534148 |
| GO:0034260~negative regulation of GTPase activity | 6 | 0.77720207 | 0.02776364 |
| GO:0007049~cell cycle | 16 | 2.07253886 | 0.02836621 |
| GO:1901216~positive regulation of neuron death | 4 | 0.51813472 | 0.02854163 |
| GO:0006516~glycoprotein catabolic process | 3 | 0.38860104 | 0.02937543 |
| GO:0043406~positive regulation of MAP kinase activity | 7 | 0.90673575 | 0.03005401 |
| GO:1900034~regulation of cellular response to heat | 8 | 1.03626943 | 0.03020817 |
| GO:2000379~positive regulation of reactive oxygen species metabolic process | 5 | 0.64766839 | 0.03050602 |
| GO:0050679~positive regulation of epithelial cell proliferation | 7 | 0.90673575 | 0.03230733 |
| GO:0007043~cell-cell junction assembly | 4 | 0.51813472 | 0.03325646 |
| GO:0031668~cellular response to extracellular stimulus | 4 | 0.51813472 | 0.03325646 |
| GO:0031667~response to nutrient levels | 5 | 0.64766839 | 0.03395246 |
| GO:0071222~cellular response to lipopolysaccharide | 10 | 1.29533679 | 0.03745367 |
| GO:0046034~ATP metabolic process | 5 | 0.64766839 | 0.0376161 |
| GO:0033601~positive regulation of mammary gland epithelial cell proliferation | 3 | 0.38860104 | 0.03814303 |
| GO:0050773~regulation of dendrite development | 3 | 0.38860104 | 0.03814303 |
| GO:0010544~negative regulation of platelet activation | 3 | 0.38860104 | 0.03814303 |
| GO:0008283~cell proliferation | 23 | 2.97927461 | 0.03819483 |
| GO:0021772~olfactory bulb development | 4 | 0.51813472 | 0.0383491 |
| GO:1902236~negative regulation of endoplasmic reticulum stress-induced intrinsic apoptotic signaling pathway | 4 | 0.51813472 | 0.0383491 |
| GO:0032956~regulation of actin cytoskeleton organization | 6 | 0.77720207 | 0.03889182 |
| GO:0071364~cellular response to epidermal growth factor stimulus | 5 | 0.64766839 | 0.04149761 |
| GO:1902042~negative regulation of extrinsic apoptotic signaling pathway via death domain receptors | 5 | 0.64766839 | 0.04149761 |
| GO:0010862~positive regulation of pathway-restricted SMAD protein phosphorylation | 6 | 0.77720207 | 0.04204182 |
| GO:0007420~brain development | 14 | 1.8134715 | 0.04219075 |
| GO:0035023~regulation of Rho protein signal transduction | 8 | 1.03626943 | 0.04325901 |
| GO:0021762~substantia nigra development | 6 | 0.77720207 | 0.0453413 |
| GO:0042981~regulation of apoptotic process | 15 | 1.94300518 | 0.04764331 |
| GO:1904754~positive regulation of vascular associated smooth muscle cell migration | 3 | 0.38860104 | 0.04776315 |
| GO:0000132~establishment of mitotic spindle orientation | 4 | 0.51813472 | 0.0496436 |
| GO:0042730~fibrinolysis | 4 | 0.51813472 | 0.0496436 |
| GO:0071902~positive regulation of protein serine/threonine kinase activity | 5 | 0.64766839 | 0.04991405 |
| GO:0072593~reactive oxygen species metabolic process | 5 | 0.64766839 | 0.04991405 |
