# Supplementary material for: Analysis of METTL3 and METTL14 in hepatocellular carcinoma
Source: Aging (Albany NY). 2020 Nov 6;12(21):21638–59. doi: 10.18632/aging.103959 (PMC7695415; doi:10.18632/aging.103959)
Supplement: Supplementary Table 2 [file aging-12-103959-s002..docx]

**Supplementary Table 2. GO analysis of DEGs regulated by METTL3.**

| **Term** | **Count** | **%** | **PValue** |
| --- | --- | --- | --- |
| GO:0030198~extracellular matrix organization | 34 | 1.95627158 | 2.21E-04 |
| GO:0045429~positive regulation of nitric oxide biosynthetic process | 13 | 0.74798619 | 2.43E-04 |
| GO:0001974~blood vessel remodeling | 11 | 0.63291139 | 2.90E-04 |
| GO:0016311~dephosphorylation | 19 | 1.09321059 | 4.29E-04 |
| GO:0006865~amino acid transport | 11 | 0.63291139 | 6.47E-04 |
| GO:0048172~regulation of short-term neuronal synaptic plasticity | 7 | 0.4027618 | 7.49E-04 |
| GO:0010628~positive regulation of gene expression | 40 | 2.30149597 | 8.25E-04 |
| GO:0043065~positive regulation of apoptotic process | 44 | 2.53164557 | 0.00101471 |
| GO:0030199~collagen fibril organization | 11 | 0.63291139 | 0.00161881 |
| GO:0043410~positive regulation of MAPK cascade | 17 | 0.97813579 | 0.00167349 |
| GO:0014047~glutamate secretion | 9 | 0.51783659 | 0.00223315 |
| GO:0006974~cellular response to DNA damage stimulus | 32 | 1.84119678 | 0.00262722 |
| GO:0030855~epithelial cell differentiation | 15 | 0.86306099 | 0.00282001 |
| GO:0007040~lysosome organization | 10 | 0.57537399 | 0.00330346 |
| GO:0055072~iron ion homeostasis | 9 | 0.51783659 | 0.00358952 |
| GO:0045995~regulation of embryonic development | 6 | 0.3452244 | 0.00372129 |
| GO:0042493~response to drug | 42 | 2.41657077 | 0.00405762 |
| GO:0071456~cellular response to hypoxia | 18 | 1.03567319 | 0.00408963 |
| GO:0071222~cellular response to lipopolysaccharide | 20 | 1.15074799 | 0.00451328 |
| GO:0008637~apoptotic mitochondrial changes | 7 | 0.4027618 | 0.00460713 |
| GO:0000188~inactivation of MAPK activity | 8 | 0.46029919 | 0.0047357 |
| GO:0030206~chondroitin sulfate biosynthetic process | 8 | 0.46029919 | 0.0047357 |
| GO:0010838~positive regulation of keratinocyte proliferation | 5 | 0.287687 | 0.00527311 |
| GO:0007623~circadian rhythm | 15 | 0.86306099 | 0.00541395 |
| GO:0048661~positive regulation of smooth muscle cell proliferation | 13 | 0.74798619 | 0.00548739 |
| GO:0045668~negative regulation of osteoblast differentiation | 10 | 0.57537399 | 0.00585261 |
| GO:0042127~regulation of cell proliferation | 28 | 1.61104718 | 0.00634035 |
| GO:0001938~positive regulation of endothelial cell proliferation | 14 | 0.80552359 | 0.00666132 |
| GO:0051402~neuron apoptotic process | 9 | 0.51783659 | 0.00672077 |
| GO:0009636~response to toxic substance | 16 | 0.92059839 | 0.00697879 |
| GO:0033627~cell adhesion mediated by integrin | 6 | 0.3452244 | 0.00748149 |
| GO:0060349~bone morphogenesis | 8 | 0.46029919 | 0.00748985 |
| GO:1900182~positive regulation of protein localization to nucleus | 7 | 0.4027618 | 0.00791083 |
| GO:0007155~cell adhesion | 57 | 3.27963176 | 0.00800048 |
| GO:0032060~bleb assembly | 5 | 0.287687 | 0.00818028 |
| GO:0097190~apoptotic signaling pathway | 14 | 0.80552359 | 0.00850199 |
| GO:0016049~cell growth | 12 | 0.69044879 | 0.00881798 |
| GO:0007528~neuromuscular junction development | 8 | 0.46029919 | 0.00924209 |
| GO:0010212~response to ionizing radiation | 11 | 0.63291139 | 0.00934938 |
| GO:0010613~positive regulation of cardiac muscle hypertrophy | 6 | 0.3452244 | 0.01010426 |
| GO:0007507~heart development | 27 | 1.55350978 | 0.01025487 |
| GO:0018406~protein C-linked glycosylation via 2'-alpha-mannosyl-L-tryptophan | 4 | 0.2301496 | 0.01114817 |
| GO:0043524~negative regulation of neuron apoptotic process | 21 | 1.20828539 | 0.01162125 |
| GO:0070542~response to fatty acid | 6 | 0.3452244 | 0.01329382 |
| GO:0007179~transforming growth factor beta receptor signaling pathway | 16 | 0.92059839 | 0.01427628 |
| GO:0060048~cardiac muscle contraction | 10 | 0.57537399 | 0.01520344 |
| GO:0045765~regulation of angiogenesis | 8 | 0.46029919 | 0.01628301 |
| GO:0090399~replicative senescence | 5 | 0.287687 | 0.01671826 |
| GO:0008286~insulin receptor signaling pathway | 14 | 0.80552359 | 0.01823305 |
| GO:0046069~cGMP catabolic process | 4 | 0.2301496 | 0.01824034 |
| GO:0006977~DNA damage response, signal transduction by p53 class mediator resulting in cell cycle arrest | 12 | 0.69044879 | 0.01856883 |
| GO:0030308~negative regulation of cell growth | 19 | 1.09321059 | 0.0191467 |
| GO:0050873~brown fat cell differentiation | 8 | 0.46029919 | 0.01929981 |
| GO:0072659~protein localization to plasma membrane | 12 | 0.69044879 | 0.02076322 |
| GO:0042981~regulation of apoptotic process | 29 | 1.66858458 | 0.02119888 |
| GO:0006520~cellular amino acid metabolic process | 9 | 0.51783659 | 0.02152348 |
| GO:1903984~positive regulation of TRAIL-activated apoptotic signaling pathway | 3 | 0.1726122 | 0.02192334 |
| GO:0007181~transforming growth factor beta receptor complex assembly | 3 | 0.1726122 | 0.02192334 |
| GO:0038145~macrophage colony-stimulating factor signaling pathway | 3 | 0.1726122 | 0.02192334 |
| GO:0002931~response to ischemia | 8 | 0.46029919 | 0.02268648 |
| GO:0051491~positive regulation of filopodium assembly | 7 | 0.4027618 | 0.02299064 |
| GO:0000732~strand displacement | 7 | 0.4027618 | 0.02299064 |
| GO:0006915~apoptotic process | 65 | 3.73993096 | 0.02317932 |
| GO:0007517~muscle organ development | 15 | 0.86306099 | 0.02339366 |
| GO:0007565~female pregnancy | 15 | 0.86306099 | 0.02339366 |
| GO:0051726~regulation of cell cycle | 19 | 1.09321059 | 0.02400624 |
| GO:0032922~circadian regulation of gene expression | 11 | 0.63291139 | 0.0259644 |
| GO:0050731~positive regulation of peptidyl-tyrosine phosphorylation | 14 | 0.80552359 | 0.02666784 |
| GO:0071157~negative regulation of cell cycle arrest | 6 | 0.3452244 | 0.02671281 |
| GO:0031572~G2 DNA damage checkpoint | 6 | 0.3452244 | 0.02671281 |
| GO:0016081~synaptic vesicle docking | 4 | 0.2301496 | 0.02729567 |
| GO:0035590~purinergic nucleotide receptor signaling pathway | 4 | 0.2301496 | 0.02729567 |
| GO:0050650~chondroitin sulfate proteoglycan biosynthetic process | 4 | 0.2301496 | 0.02729567 |
| GO:0001955~blood vessel maturation | 4 | 0.2301496 | 0.02729567 |
| GO:0035234~ectopic germ cell programmed cell death | 4 | 0.2301496 | 0.02729567 |
| GO:0060100~positive regulation of phagocytosis, engulfment | 4 | 0.2301496 | 0.02729567 |
| GO:0010575~positive regulation of vascular endothelial growth factor production | 7 | 0.4027618 | 0.02741944 |
| GO:0001541~ovarian follicle development | 9 | 0.51783659 | 0.02827943 |
| GO:0016477~cell migration | 24 | 1.38089758 | 0.02881607 |
| GO:0070373~negative regulation of ERK1 and ERK2 cascade | 11 | 0.63291139 | 0.02898311 |
| GO:0030889~negative regulation of B cell proliferation | 5 | 0.287687 | 0.02934348 |
| GO:2001241~positive regulation of extrinsic apoptotic signaling pathway in absence of ligand | 5 | 0.287687 | 0.02934348 |
| GO:0031103~axon regeneration | 5 | 0.287687 | 0.02934348 |
| GO:0086091~regulation of heart rate by cardiac conduction | 8 | 0.46029919 | 0.03064343 |
| GO:0000731~DNA synthesis involved in DNA repair | 8 | 0.46029919 | 0.03064343 |
| GO:0042594~response to starvation | 8 | 0.46029919 | 0.03064343 |
| GO:0050918~positive chemotaxis | 8 | 0.46029919 | 0.03064343 |
| GO:0072593~reactive oxygen species metabolic process | 8 | 0.46029919 | 0.03064343 |
| GO:0032496~response to lipopolysaccharide | 23 | 1.32336018 | 0.03099505 |
| GO:0070588~calcium ion transmembrane transport | 18 | 1.03567319 | 0.03178092 |
| GO:0051881~regulation of mitochondrial membrane potential | 7 | 0.4027618 | 0.0323769 |
| GO:0086010~membrane depolarization during action potential | 7 | 0.4027618 | 0.0323769 |
| GO:0007269~neurotransmitter secretion | 10 | 0.57537399 | 0.03255518 |
| GO:0002028~regulation of sodium ion transport | 6 | 0.3452244 | 0.03258843 |
| GO:0033198~response to ATP | 6 | 0.3452244 | 0.03258843 |
| GO:0000082~G1/S transition of mitotic cell cycle | 16 | 0.92059839 | 0.03351157 |
| GO:0045669~positive regulation of osteoblast differentiation | 11 | 0.63291139 | 0.03575047 |
| GO:0034097~response to cytokine | 10 | 0.57537399 | 0.03640544 |
| GO:0006198~cAMP catabolic process | 5 | 0.287687 | 0.0372933 |
| GO:0001822~kidney development | 14 | 0.80552359 | 0.03766122 |
| GO:0008354~germ cell migration | 4 | 0.2301496 | 0.03830695 |
| GO:2001243~negative regulation of intrinsic apoptotic signaling pathway | 6 | 0.3452244 | 0.03920819 |
| GO:0010719~negative regulation of epithelial to mesenchymal transition | 6 | 0.3452244 | 0.03920819 |
| GO:0001954~positive regulation of cell-matrix adhesion | 6 | 0.3452244 | 0.03920819 |
| GO:0061337~cardiac conduction | 9 | 0.51783659 | 0.04094959 |
| GO:0032755~positive regulation of interleukin-6 production | 9 | 0.51783659 | 0.04094959 |
| GO:0006021~inositol biosynthetic process | 3 | 0.1726122 | 0.04129346 |
| GO:0050689~negative regulation of defense response to virus by host | 3 | 0.1726122 | 0.04129346 |
| GO:0038124~toll-like receptor TLR6:TLR2 signaling pathway | 3 | 0.1726122 | 0.04129346 |
| GO:0032386~regulation of intracellular transport | 3 | 0.1726122 | 0.04129346 |
| GO:0043276~anoikis | 3 | 0.1726122 | 0.04129346 |
| GO:0045217~cell-cell junction maintenance | 3 | 0.1726122 | 0.04129346 |
| GO:0071726~cellular response to diacyl bacterial lipopeptide | 3 | 0.1726122 | 0.04129346 |
| GO:1904491~protein localization to ciliary transition zone | 3 | 0.1726122 | 0.04129346 |
| GO:0048146~positive regulation of fibroblast proliferation | 10 | 0.57537399 | 0.04501462 |
| GO:0071260~cellular response to mechanical stimulus | 12 | 0.69044879 | 0.04561142 |
| GO:0030900~forebrain development | 9 | 0.51783659 | 0.04589341 |
| GO:0035994~response to muscle stretch | 5 | 0.287687 | 0.04635631 |
| GO:0048566~embryonic digestive tract development | 5 | 0.287687 | 0.04635631 |
| GO:0008206~bile acid metabolic process | 5 | 0.287687 | 0.04635631 |
| GO:0035269~protein O-linked mannosylation | 5 | 0.287687 | 0.04635631 |
| GO:0008209~androgen metabolic process | 5 | 0.287687 | 0.04635631 |
| GO:0010744~positive regulation of macrophage derived foam cell differentiation | 5 | 0.287687 | 0.04635631 |
| GO:0055114~oxidation-reduction process | 65 | 3.73993096 | 0.05061517 |
| GO:0006470~protein dephosphorylation | 18 | 1.03567319 | 0.05071141 |
| GO:0031100~organ regeneration | 9 | 0.51783659 | 0.05121201 |
| GO:0071230~cellular response to amino acid stimulus | 9 | 0.51783659 | 0.05121201 |
| GO:0045080~positive regulation of chemokine biosynthetic process | 4 | 0.2301496 | 0.05121838 |
| GO:0045475~locomotor rhythm | 4 | 0.2301496 | 0.05121838 |
| GO:0042118~endothelial cell activation | 4 | 0.2301496 | 0.05121838 |
| GO:0033327~Leydig cell differentiation | 4 | 0.2301496 | 0.05121838 |
| GO:0016572~histone phosphorylation | 4 | 0.2301496 | 0.05121838 |
| GO:0002377~immunoglobulin production | 4 | 0.2301496 | 0.05121838 |
| GO:0035335~peptidyl-tyrosine dephosphorylation | 15 | 0.86306099 | 0.05175329 |
| GO:0060337~type I interferon signaling pathway | 11 | 0.63291139 | 0.05241508 |
| GO:0035725~sodium ion transmembrane transport | 12 | 0.69044879 | 0.05409557 |
| GO:0005975~carbohydrate metabolic process | 23 | 1.32336018 | 0.05418076 |
| GO:0045737~positive regulation of cyclin-dependent protein serine/threonine kinase activity | 6 | 0.3452244 | 0.05473439 |
| GO:0050680~negative regulation of epithelial cell proliferation | 10 | 0.57537399 | 0.05488456 |
| GO:0060749~mammary gland alveolus development | 5 | 0.287687 | 0.05652939 |
| GO:2000811~negative regulation of anoikis | 5 | 0.287687 | 0.05652939 |
| GO:0090201~negative regulation of release of cytochrome c from mitochondria | 5 | 0.287687 | 0.05652939 |
| GO:0030224~monocyte differentiation | 5 | 0.287687 | 0.05652939 |
| GO:0010460~positive regulation of heart rate | 5 | 0.287687 | 0.05652939 |
| GO:0001525~angiogenesis | 28 | 1.61104718 | 0.05658092 |
| GO:0006493~protein O-linked glycosylation | 7 | 0.4027618 | 0.05780762 |
| GO:0035556~intracellular signal transduction | 46 | 2.64672037 | 0.05883485 |
| GO:0071356~cellular response to tumor necrosis factor | 16 | 0.92059839 | 0.05896005 |
| GO:0009615~response to virus | 16 | 0.92059839 | 0.05896005 |
| GO:0007417~central nervous system development | 17 | 0.97813579 | 0.06194593 |
| GO:0014070~response to organic cyclic compound | 9 | 0.51783659 | 0.06299853 |
| GO:0071560~cellular response to transforming growth factor beta stimulus | 9 | 0.51783659 | 0.06299853 |
| GO:2000134~negative regulation of G1/S transition of mitotic cell cycle | 6 | 0.3452244 | 0.06365081 |
| GO:0061156~pulmonary artery morphogenesis | 3 | 0.1726122 | 0.0648465 |
| GO:0030204~chondroitin sulfate metabolic process | 3 | 0.1726122 | 0.0648465 |
| GO:0070257~positive regulation of mucus secretion | 3 | 0.1726122 | 0.0648465 |
| GO:1990441~negative regulation of transcription from RNA polymerase II promoter in response to endoplasmic reticulum stress | 3 | 0.1726122 | 0.0648465 |
| GO:0098735~positive regulation of the force of heart contraction | 3 | 0.1726122 | 0.0648465 |
| GO:0008285~negative regulation of cell proliferation | 45 | 2.58918297 | 0.06537547 |
| GO:0045747~positive regulation of Notch signaling pathway | 7 | 0.4027618 | 0.06561232 |
| GO:0010634~positive regulation of epithelial cell migration | 7 | 0.4027618 | 0.06561232 |
| GO:0070059~intrinsic apoptotic signaling pathway in response to endoplasmic reticulum stress | 7 | 0.4027618 | 0.06561232 |
| GO:0048703~embryonic viscerocranium morphogenesis | 4 | 0.2301496 | 0.06593724 |
| GO:0050927~positive regulation of positive chemotaxis | 4 | 0.2301496 | 0.06593724 |
| GO:0014075~response to amine | 4 | 0.2301496 | 0.06593724 |
| GO:0045446~endothelial cell differentiation | 4 | 0.2301496 | 0.06593724 |
| GO:0045944~positive regulation of transcription from RNA polymerase II promoter | 101 | 5.81127733 | 0.06772679 |
| GO:0031668~cellular response to extracellular stimulus | 5 | 0.287687 | 0.06779549 |
| GO:0061436~establishment of skin barrier | 5 | 0.287687 | 0.06779549 |
| GO:0051412~response to corticosterone | 5 | 0.287687 | 0.06779549 |
| GO:0007062~sister chromatid cohesion | 15 | 0.86306099 | 0.06789861 |
| GO:0010506~regulation of autophagy | 9 | 0.51783659 | 0.06947517 |
| GO:0045740~positive regulation of DNA replication | 8 | 0.46029919 | 0.07232365 |
| GO:0002053~positive regulation of mesenchymal cell proliferation | 6 | 0.3452244 | 0.0733318 |
| GO:0030182~neuron differentiation | 14 | 0.80552359 | 0.07334204 |
| GO:0045494~photoreceptor cell maintenance | 7 | 0.4027618 | 0.07400169 |
| GO:0097192~extrinsic apoptotic signaling pathway in absence of ligand | 7 | 0.4027618 | 0.07400169 |
| GO:0050919~negative chemotaxis | 7 | 0.4027618 | 0.07400169 |
| GO:0051092~positive regulation of NF-kappaB transcription factor activity | 18 | 1.03567319 | 0.0764338 |
| GO:0051091~positive regulation of sequence-specific DNA binding transcription factor activity | 15 | 0.86306099 | 0.0770987 |
| GO:0001666~response to hypoxia | 22 | 1.26582278 | 0.07899131 |
| GO:0046716~muscle cell cellular homeostasis | 5 | 0.287687 | 0.0801252 |
| GO:1904659~glucose transmembrane transport | 5 | 0.287687 | 0.0801252 |
| GO:0045766~positive regulation of angiogenesis | 16 | 0.92059839 | 0.08025756 |
| GO:0032228~regulation of synaptic transmission, GABAergic | 4 | 0.2301496 | 0.08234356 |
| GO:2000505~regulation of energy homeostasis | 4 | 0.2301496 | 0.08234356 |
| GO:0060707~trophoblast giant cell differentiation | 4 | 0.2301496 | 0.08234356 |
| GO:0001706~endoderm formation | 4 | 0.2301496 | 0.08234356 |
| GO:0030208~dermatan sulfate biosynthetic process | 4 | 0.2301496 | 0.08234356 |
| GO:0036499~PERK-mediated unfolded protein response | 4 | 0.2301496 | 0.08234356 |
| GO:0055119~relaxation of cardiac muscle | 4 | 0.2301496 | 0.08234356 |
| GO:0071498~cellular response to fluid shear stress | 4 | 0.2301496 | 0.08234356 |
| GO:0033572~transferrin transport | 7 | 0.4027618 | 0.08297236 |
| GO:0051281~positive regulation of release of sequestered calcium ion into cytosol | 6 | 0.3452244 | 0.08376643 |
| GO:0015721~bile acid and bile salt transport | 6 | 0.3452244 | 0.08376643 |
| GO:0006400~tRNA modification | 6 | 0.3452244 | 0.08376643 |
| GO:0031032~actomyosin structure organization | 6 | 0.3452244 | 0.08376643 |
| GO:0032092~positive regulation of protein binding | 10 | 0.57537399 | 0.08533043 |
| GO:0001523~retinoid metabolic process | 10 | 0.57537399 | 0.08533043 |
| GO:0006635~fatty acid beta-oxidation | 8 | 0.46029919 | 0.088414 |
| GO:0007098~centrosome cycle | 3 | 0.1726122 | 0.09169567 |
| GO:0003416~endochondral bone growth | 3 | 0.1726122 | 0.09169567 |
| GO:0050957~equilibrioception | 3 | 0.1726122 | 0.09169567 |
| GO:0003223~ventricular compact myocardium morphogenesis | 3 | 0.1726122 | 0.09169567 |
| GO:0010694~positive regulation of alkaline phosphatase activity | 3 | 0.1726122 | 0.09169567 |
| GO:0043619~regulation of transcription from RNA polymerase II promoter in response to oxidative stress | 3 | 0.1726122 | 0.09169567 |
| GO:0090527~actin filament reorganization | 3 | 0.1726122 | 0.09169567 |
| GO:0061002~negative regulation of dendritic spine morphogenesis | 3 | 0.1726122 | 0.09169567 |
| GO:0060426~lung vasculature development | 3 | 0.1726122 | 0.09169567 |
| GO:0072719~cellular response to cisplatin | 3 | 0.1726122 | 0.09169567 |
| GO:0035264~multicellular organism growth | 12 | 0.69044879 | 0.09176324 |
| GO:0060395~SMAD protein signal transduction | 10 | 0.57537399 | 0.09242638 |
| GO:0001934~positive regulation of protein phosphorylation | 17 | 0.97813579 | 0.09244176 |
| GO:0050714~positive regulation of protein secretion | 7 | 0.4027618 | 0.09251755 |
| GO:0022904~respiratory electron transport chain | 5 | 0.287687 | 0.09347829 |
| GO:0003281~ventricular septum development | 6 | 0.3452244 | 0.09493806 |
| GO:0018146~keratan sulfate biosynthetic process | 6 | 0.3452244 | 0.09493806 |
| GO:0001707~mesoderm formation | 6 | 0.3452244 | 0.09493806 |
| GO:0051260~protein homooligomerization | 22 | 1.26582278 | 0.09923697 |
