# Supplementary material for: Analysis of METTL3 and METTL14 in hepatocellular carcinoma
Source: Aging (Albany NY). 2020 Nov 6;12(21):21638–59. doi: 10.18632/aging.103959 (PMC7695415; doi:10.18632/aging.103959)
Supplement: Supplementary Tables 3, 4, 5 and 6 [file aging-12-103959-s003..pdf]

SUPPLEMENTARY TABLES

Supplementary Table 3. The correlations between the expression of hub genes and METTL3 expression in HCC.

Gene	R	P value	Gene	R	P value
ZWINT	0.51	<0.001	CLSPN	0.5	<0.001
MYLIP	0.32	<0.001	TIMELESS	0.56	<0.001
FANCI	0.43	<0.001	MLEC	0.49	<0.001
EXO1	0.51	<0.001	DTL	0.47	<0.001
HGSNAT	0.31	<0.001	CBLB	0.43	<0.001
BTBD1	0.33	<0.001	SPSB2	0.39	<0.001
KNTC1	0.59	<0.001	PLAUR	0.25	<0.001
DET1	0.42	<0.001	MELK	0.53	<0.001
FBXO2	-0.1	0.05	KBTBD6	0.45	<0.001
SLC2A3	0.11	0.039	ASPM	0.51	<0.001
KCNAB2	0.024	0.65	KLHL22	0.45	<0.001
FBXW4	0.36	<0.001	ALDH3B1	0.23	<0.001
HERC6	0.078	0.13	TSPAN14	0.43	<0.001
STOM	0.019	0.17	ASF1B	0.4	<0.001
UBOX5	0.48	<0.001	BRCA1	0.46	<0.001

Supplementary Table 4. The associations between the expression of hub genes and overall survival of HCC patients.

Gene	HR (95%CI)	P value	Gene	HR (95%CI)	P value
ZWINT	2.36 (1.66-3.36)	<0.001	CLSPN	2.26 (1.6-3.19)	<0.001
MYLIP	1.41 (0.99-2.01)	0.059	TIMELESS	1.63 (1.15-2.3)	0.0053
FANCI	2.11 (1.43-3.13)	<0.001	MLEC	1.31 (0.89-1.92)	0.17
EXO1	2.3 (1.63-3.26)	<0.001	DTL	1.89 (1.33-2.69)	<0.001
HGSNAT	0.67 (0.47-0.96)	0.029	CBLB	1.2 (0.85-1.69)	0.3
BTBD1	0.69 (0.46-1.03)	0.07	SPSB2	1.71 (1.2-2.43)	0.0026
KNTC1	1.96 (1.36-2.81)	<0.001	PLAUR	1.99 (1.25-3.18)	0.0032
DET1	0.65 (0.45-0.93)	0.018	MELK	2.22 (1.5-3.27)	<0.001
FBXO2	0.62 (0.044-0.89)	0.0081	KBTBD6	0.58 (0.41-0.83)	0.0021
SLC2A3	0.8 (0.54-1.18)	0.25	ASPM	2.01 (1.39-2.92)	<0.001
KCNAB2	1.32 (0.88-1.97)	0.17	KLHL22	0.86 (0.6-1.22)	0.4
FBXW4	0.62 (0.44-0.88)	0.0067	ALDH3B1	1.53 (1.08-2.18)	0.017
HERC6	0.69 (0.48-0.99)	0.044	TSPAN14	0.69 (0.48-1)	0.047
STOM	0.52 (0.35-0.78)	0.0015	ASF1B	1.71 (1.21-2.42)	0.002
UBOX5	0.73 (0.52-1.05)	0.077	BRCA1	1.82 (1.26-2.61)	0.0011

Supplementary Table 5. The correlations between the expression of hub genes and METTL14 expression in HCC.

Gene	R	P value	Gene	R	P value
FBXO2	0.032	0.54	WDR43	0.51	<0.001
FBXW2	0.58	<0.001	SPSB1	0.24	<0.001
ASB13	-0.1	0.055	RRP1B	0.52	<0.001
FBXL15	-0.016	0.75	NOP56	-0.077	0.14
VPRBP	0.73	<0.001	FBXO10	0.26	<0.001
NOL10	0.44	<0.001	ANKRD9	0.22	<0.001
NOM1	0.48	<0.001	BMP4	0.18	<0.001
POLR3B	0.55	<0.001	UTP3	0.56	<0.001
LGALS1	-0.099	0.058	IGFBP1	0.084	0.11
WDR75	0.32	<0.001	STC2	0.093	0.075
UTP15	0.59	<0.001	CALU	0.34	<0.001
RNF4	0.53	<0.001	PAK1IP1	0.35	<0.001
FBXW5	0.26	<0.001	RSL1D1	0.46	<0.001
RBM19	0.22	<0.001	WFS1	0.12	0.023
SIAH2	0.26	<0.001	LAMC1	0.38	<0.001

Supplementary Table 6. The associations between the expression of hub genes and overall survival of HCC patients.

Gene	HR (95%CI)	P value	Gene	HR (95%CI)	P value
FBXO2	0.62 (0.44-0.89)	0.0081	WDR43	1.64 (1.15-2.33)	0.0055
FBXW2	0.7 (0.49-0.99)	0.045	SPSB1	0.78 (0.55-1.1)	0.16
ASB13	0.79 (0.55-1.16)	0.23	RRP1B	0.81 (0.54-1.22)	0.31
FBXL15	0.61 (0.42-0.89)	0.0091	NOP56	2.33 (1.65-3.3)	<0.001
VPRBP	0.78 (0.56-1.11)	0.17	FBXO10	1.46 (1.01-2.12)	0.041
NOL10	1.88 (1.32-2.68)	<0.001	ANKRD9	0.65 (0.46-0.93)	0.017
NOM1	0.79 (0.55-1.11)	0.17	BMP4	1.38 (0.96-1.99)	0.082
POLR3B	0.78 (0.54-1.12)	0.18	UTP3	0.8 (0.57-1.14)	0.22
LGALS1	1.56 (1.08-2.25)	0.018	IGFBP1	0.73 (0.51-1.03)	0.072
WDR75	2.09 (1.45-3.02)	<0.001	STC2	1.95 (1.38-2.75)	<0.001
UTP15	1.18 (0.8-1.74)	0.4	CALU	1.85 (1.28-2.68)	<0.001
RNF4	1.31 (0.92-1.87)	0.13	PAK1IP1	1.98 (1.35-2.91)	<0.001
FBXW5	0.88 (0.62-1.25)	0.47	RSL1D1	1.33 (0.94-1.88)	0.1
RBM19	2.03 (1.42-2.89)	<0.001	WFS1	1.45 (0.95-2.2)	0.081
SIAH2	0.54 (0.38-0.78)	<0.001	LAMC1	1.43 (0.98-2.09)	0.062
