# Supplementary material for: Analysis of METTL3 and METTL14 in hepatocellular carcinoma
Source: Aging (Albany NY). 2020 Nov 6;12(21):21638–59. doi: 10.18632/aging.103959 (PMC7695415; doi:10.18632/aging.103959)
Supplement: Supplementary Table 7 [file aging-12-103959-s004..docx]

**Supplementary Table 7. GO analysis of TEGs regulated by METTL3.**

| **Term** | **Count** | **%** | **PValue** |
| --- | --- | --- | --- |
| GO:0032200~telomere organization | 12 | 0.64585576 | 1.08E-05 |
| GO:0006334~nucleosome assembly | 27 | 1.45317546 | 1.85E-05 |
| GO:0008286~insulin receptor signaling pathway | 20 | 1.07642626 | 5.04E-05 |
| GO:0000183~chromatin silencing at rDNA | 13 | 0.69967707 | 6.31E-05 |
| GO:0006335~DNA replication-dependent nucleosome assembly | 12 | 0.64585576 | 6.98E-05 |
| GO:0007219~Notch signaling pathway | 25 | 1.34553283 | 8.31E-05 |
| GO:0006366~transcription from RNA polymerase II promoter | 73 | 3.92895587 | 1.17E-04 |
| GO:0006687~glycosphingolipid metabolic process | 14 | 0.75349839 | 1.21E-04 |
| GO:0060337~type I interferon signaling pathway | 17 | 0.91496233 | 1.40E-04 |
| GO:0019083~viral transcription | 24 | 1.29171152 | 1.50E-04 |
| GO:0000122~negative regulation of transcription from RNA polymerase II promoter | 95 | 5.11302476 | 1.78E-04 |
| GO:0006413~translational initiation | 27 | 1.45317546 | 2.22E-04 |
| GO:0045815~positive regulation of gene expression, epigenetic | 16 | 0.86114101 | 3.30E-04 |
| GO:0001649~osteoblast differentiation | 22 | 1.18406889 | 3.63E-04 |
| GO:0045944~positive regulation of transcription from RNA polymerase II promoter | 121 | 6.5123789 | 3.67E-04 |
| GO:0045814~negative regulation of gene expression, epigenetic | 14 | 0.75349839 | 3.83E-04 |
| GO:0045892~negative regulation of transcription, DNA-templated | 69 | 3.71367061 | 4.21E-04 |
| GO:0006364~rRNA processing | 36 | 1.93756728 | 4.30E-04 |
| GO:2001244~positive regulation of intrinsic apoptotic signaling pathway | 11 | 0.59203445 | 4.78E-04 |
| GO:0001836~release of cytochrome c from mitochondria | 9 | 0.48439182 | 6.31E-04 |
| GO:0006614~SRP-dependent cotranslational protein targeting to membrane | 20 | 1.07642626 | 6.70E-04 |
| GO:0033572~transferrin transport | 11 | 0.59203445 | 8.02E-04 |
| GO:0044267~cellular protein metabolic process | 23 | 1.2378902 | 8.39E-04 |
| GO:0016032~viral process | 45 | 2.4219591 | 8.96E-04 |
| GO:0051290~protein heterotetramerization | 12 | 0.64585576 | 9.94E-04 |
| GO:0045893~positive regulation of transcription, DNA-templated | 69 | 3.71367061 | 9.94E-04 |
| GO:0042475~odontogenesis of dentin-containing tooth | 14 | 0.75349839 | 0.0010229 |
| GO:0098609~cell-cell adhesion | 41 | 2.20667384 | 0.00141267 |
| GO:0031175~neuron projection development | 20 | 1.07642626 | 0.00146028 |
| GO:0060333~interferon-gamma-mediated signaling pathway | 16 | 0.86114101 | 0.00149522 |
| GO:0006336~DNA replication-independent nucleosome assembly | 9 | 0.48439182 | 0.0015713 |
| GO:0051607~defense response to virus | 28 | 1.50699677 | 0.00183285 |
| GO:0070059~intrinsic apoptotic signaling pathway in response to endoplasmic reticulum stress | 10 | 0.53821313 | 0.00208245 |
| GO:0000184~nuclear-transcribed mRNA catabolic process, nonsense-mediated decay | 22 | 1.18406889 | 0.0022201 |
| GO:0018279~protein N-linked glycosylation via asparagine | 11 | 0.59203445 | 0.00243989 |
| GO:0051603~proteolysis involved in cellular protein catabolic process | 12 | 0.64585576 | 0.00317085 |
| GO:0010971~positive regulation of G2/M transition of mitotic cell cycle | 7 | 0.37674919 | 0.00390493 |
| GO:0007050~cell cycle arrest | 24 | 1.29171152 | 0.00399297 |
| GO:0051301~cell division | 48 | 2.58342304 | 0.00406131 |
| GO:0002474~antigen processing and presentation of peptide antigen via MHC class I | 9 | 0.48439182 | 0.00424904 |
| GO:0045429~positive regulation of nitric oxide biosynthetic process | 11 | 0.59203445 | 0.00430172 |
| GO:0030335~positive regulation of cell migration | 29 | 1.56081808 | 0.00445777 |
| GO:0048010~vascular endothelial growth factor receptor signaling pathway | 15 | 0.8073197 | 0.00472056 |
| GO:0031047~gene silencing by RNA | 20 | 1.07642626 | 0.00496045 |
| GO:0032508~DNA duplex unwinding | 11 | 0.59203445 | 0.00512423 |
| GO:0032481~positive regulation of type I interferon production | 12 | 0.64585576 | 0.0052015 |
| GO:0060716~labyrinthine layer blood vessel development | 7 | 0.37674919 | 0.00527551 |
| GO:0050821~protein stabilization | 23 | 1.2378902 | 0.00531291 |
| GO:0001568~blood vessel development | 10 | 0.53821313 | 0.00583389 |
| GO:0001889~liver development | 15 | 0.8073197 | 0.00607935 |
| GO:0090090~negative regulation of canonical Wnt signaling pathway | 26 | 1.39935414 | 0.00627602 |
| GO:0008285~negative regulation of cell proliferation | 52 | 2.79870829 | 0.00653919 |
| GO:0030433~ER-associated ubiquitin-dependent protein catabolic process | 13 | 0.69967707 | 0.00680522 |
| GO:0003151~outflow tract morphogenesis | 11 | 0.59203445 | 0.00713478 |
| GO:0006469~negative regulation of protein kinase activity | 18 | 0.96878364 | 0.0073701 |
| GO:0006412~translation | 36 | 1.93756728 | 0.0076486 |
| GO:0051726~regulation of cell cycle | 21 | 1.13024758 | 0.00788067 |
| GO:0048146~positive regulation of fibroblast proliferation | 12 | 0.64585576 | 0.00814257 |
| GO:0045071~negative regulation of viral genome replication | 10 | 0.53821313 | 0.00830769 |
| GO:0032728~positive regulation of interferon-beta production | 8 | 0.43057051 | 0.00867366 |
| GO:1902237~positive regulation of endoplasmic reticulum stress-induced intrinsic apoptotic signaling pathway | 5 | 0.26910657 | 0.00901364 |
| GO:0006921~cellular component disassembly involved in execution phase of apoptosis | 7 | 0.37674919 | 0.00901986 |
| GO:0006888~ER to Golgi vesicle-mediated transport | 25 | 1.34553283 | 0.00957852 |
| GO:0016055~Wnt signaling pathway | 28 | 1.50699677 | 0.01032968 |
| GO:0001569~patterning of blood vessels | 8 | 0.43057051 | 0.01067981 |
| GO:0002576~platelet degranulation | 18 | 0.96878364 | 0.01089825 |
| GO:0043066~negative regulation of apoptotic process | 57 | 3.06781485 | 0.01127402 |
| GO:0043687~post-translational protein modification | 7 | 0.37674919 | 0.01147016 |
| GO:0045599~negative regulation of fat cell differentiation | 10 | 0.53821313 | 0.01150602 |
| GO:0006457~protein folding | 27 | 1.45317546 | 0.01162626 |
| GO:0032922~circadian regulation of gene expression | 12 | 0.64585576 | 0.01223416 |
| GO:0010628~positive regulation of gene expression | 36 | 1.93756728 | 0.01284719 |
| GO:0043627~response to estrogen | 13 | 0.69967707 | 0.01289424 |
| GO:0007049~cell cycle | 31 | 1.66846071 | 0.01292675 |
| GO:0048008~platelet-derived growth factor receptor signaling pathway | 8 | 0.43057051 | 0.01300249 |
| GO:0060968~regulation of gene silencing | 5 | 0.26910657 | 0.01316084 |
| GO:0046325~negative regulation of glucose import | 5 | 0.26910657 | 0.01316084 |
| GO:0034080~CENP-A containing nucleosome assembly | 10 | 0.53821313 | 0.01341304 |
| GO:0006352~DNA-templated transcription, initiation | 9 | 0.48439182 | 0.01352147 |
| GO:0032543~mitochondrial translation | 9 | 0.48439182 | 0.01352147 |
| GO:0070373~negative regulation of ERK1 and ERK2 cascade | 12 | 0.64585576 | 0.01389701 |
| GO:0038061~NIK/NF-kappaB signaling | 13 | 0.69967707 | 0.01449897 |
| GO:0007596~blood coagulation | 27 | 1.45317546 | 0.01517543 |
| GO:0071407~cellular response to organic cyclic compound | 12 | 0.64585576 | 0.01572497 |
| GO:0010468~regulation of gene expression | 17 | 0.91496233 | 0.01753239 |
| GO:0009954~proximal/distal pattern formation | 7 | 0.37674919 | 0.01770745 |
| GO:0030968~endoplasmic reticulum unfolded protein response | 10 | 0.53821313 | 0.01791433 |
| GO:0003376~sphingosine-1-phosphate signaling pathway | 5 | 0.26910657 | 0.01834774 |
| GO:0000050~urea cycle | 5 | 0.26910657 | 0.01834774 |
| GO:0042593~glucose homeostasis | 17 | 0.91496233 | 0.01913908 |
| GO:0045727~positive regulation of translation | 11 | 0.59203445 | 0.01918817 |
| GO:1904628~cellular response to phorbol 13-acetate 12-myristate | 4 | 0.21528525 | 0.01964727 |
| GO:0072577~endothelial cell apoptotic process | 4 | 0.21528525 | 0.01964727 |
| GO:0001844~protein insertion into mitochondrial membrane involved in apoptotic signaling pathway | 4 | 0.21528525 | 0.01964727 |
| GO:0048208~COPII vesicle coating | 12 | 0.64585576 | 0.01991339 |
| GO:0071158~positive regulation of cell cycle arrest | 7 | 0.37674919 | 0.02155989 |
| GO:0001756~somitogenesis | 9 | 0.48439182 | 0.02160624 |
| GO:0016477~cell migration | 25 | 1.34553283 | 0.02192017 |
| GO:0001974~blood vessel remodeling | 8 | 0.43057051 | 0.02211297 |
| GO:0000086~G2/M transition of mitotic cell cycle | 21 | 1.13024758 | 0.02223483 |
| GO:0045079~negative regulation of chemokine biosynthetic process | 3 | 0.16146394 | 0.02311522 |
| GO:0071400~cellular response to oleic acid | 3 | 0.16146394 | 0.02311522 |
| GO:0071409~cellular response to cycloheximide | 3 | 0.16146394 | 0.02311522 |
| GO:0045860~positive regulation of protein kinase activity | 10 | 0.53821313 | 0.02342385 |
| GO:0000028~ribosomal small subunit assembly | 6 | 0.32292788 | 0.02399642 |
| GO:0006878~cellular copper ion homeostasis | 5 | 0.26910657 | 0.02463838 |
| GO:0034975~protein folding in endoplasmic reticulum | 5 | 0.26910657 | 0.02463838 |
| GO:2000573~positive regulation of DNA biosynthetic process | 5 | 0.26910657 | 0.02463838 |
| GO:0002479~antigen processing and presentation of exogenous peptide antigen via MHC class I, TAP-dependent | 12 | 0.64585576 | 0.0248722 |
| GO:0071364~cellular response to epidermal growth factor stimulus | 8 | 0.43057051 | 0.02593867 |
| GO:0010718~positive regulation of epithelial to mesenchymal transition | 8 | 0.43057051 | 0.02593867 |
| GO:0006506~GPI anchor biosynthetic process | 7 | 0.37674919 | 0.02594033 |
| GO:0035264~multicellular organism growth | 14 | 0.75349839 | 0.02702135 |
| GO:0016485~protein processing | 13 | 0.69967707 | 0.02748842 |
| GO:0046825~regulation of protein export from nucleus | 4 | 0.21528525 | 0.02934584 |
| GO:1903896~positive regulation of IRE1-mediated unfolded protein response | 4 | 0.21528525 | 0.02934584 |
| GO:0060972~left/right pattern formation | 4 | 0.21528525 | 0.02934584 |
| GO:0044598~doxorubicin metabolic process | 4 | 0.21528525 | 0.02934584 |
| GO:0048513~animal organ development | 4 | 0.21528525 | 0.02934584 |
| GO:0044597~daunorubicin metabolic process | 4 | 0.21528525 | 0.02934584 |
| GO:0075713~establishment of integrated proviral latency | 4 | 0.21528525 | 0.02934584 |
| GO:0006987~activation of signaling protein activity involved in unfolded protein response | 4 | 0.21528525 | 0.02934584 |
| GO:0071157~negative regulation of cell cycle arrest | 6 | 0.32292788 | 0.02967271 |
| GO:0006120~mitochondrial electron transport, NADH to ubiquinone | 10 | 0.53821313 | 0.03004508 |
| GO:0032091~negative regulation of protein binding | 11 | 0.59203445 | 0.0306509 |
| GO:0035987~endodermal cell differentiation | 7 | 0.37674919 | 0.03087366 |
| GO:0072332~intrinsic apoptotic signaling pathway by p53 class mediator | 7 | 0.37674919 | 0.03087366 |
| GO:0008283~cell proliferation | 45 | 2.4219591 | 0.03203749 |
| GO:0043923~positive regulation by host of viral transcription | 5 | 0.26910657 | 0.03207684 |
| GO:0051016~barbed-end actin filament capping | 5 | 0.26910657 | 0.03207684 |
| GO:0048009~insulin-like growth factor receptor signaling pathway | 5 | 0.26910657 | 0.03207684 |
| GO:0045740~positive regulation of DNA replication | 9 | 0.48439182 | 0.03261813 |
| GO:0043065~positive regulation of apoptotic process | 38 | 2.0452099 | 0.03332484 |
| GO:0006302~double-strand break repair | 12 | 0.64585576 | 0.03390497 |
| GO:0016925~protein sumoylation | 18 | 0.96878364 | 0.03449473 |
| GO:0045861~negative regulation of proteolysis | 6 | 0.32292788 | 0.03612672 |
| GO:0071480~cellular response to gamma radiation | 6 | 0.32292788 | 0.03612672 |
| GO:0006974~cellular response to DNA damage stimulus | 28 | 1.50699677 | 0.03623819 |
| GO:2000145~regulation of cell motility | 7 | 0.37674919 | 0.03638107 |
| GO:0045931~positive regulation of mitotic cell cycle | 7 | 0.37674919 | 0.03638107 |
| GO:0090200~positive regulation of release of cytochrome c from mitochondria | 7 | 0.37674919 | 0.03638107 |
| GO:0034976~response to endoplasmic reticulum stress | 13 | 0.69967707 | 0.03647656 |
| GO:0007179~transforming growth factor beta receptor signaling pathway | 15 | 0.8073197 | 0.03694375 |
| GO:1904837~beta-catenin-TCF complex assembly | 9 | 0.48439182 | 0.03700786 |
| GO:0001666~response to hypoxia | 24 | 1.29171152 | 0.03769898 |
| GO:0042542~response to hydrogen peroxide | 10 | 0.53821313 | 0.03787071 |
| GO:0030307~positive regulation of cell growth | 14 | 0.75349839 | 0.03847936 |
| GO:0006979~response to oxidative stress | 17 | 0.91496233 | 0.0390694 |
| GO:0009615~response to virus | 17 | 0.91496233 | 0.0390694 |
| GO:0090307~mitotic spindle assembly | 8 | 0.43057051 | 0.0400484 |
| GO:0019985~translesion synthesis | 8 | 0.43057051 | 0.0400484 |
| GO:0006446~regulation of translational initiation | 8 | 0.43057051 | 0.0400484 |
| GO:0044458~motile cilium assembly | 5 | 0.26910657 | 0.040688 |
| GO:0033627~cell adhesion mediated by integrin | 5 | 0.26910657 | 0.040688 |
| GO:0016254~preassembly of GPI anchor in ER membrane | 5 | 0.26910657 | 0.040688 |
| GO:0007059~chromosome segregation | 12 | 0.64585576 | 0.04108086 |
| GO:0003143~embryonic heart tube morphogenesis | 4 | 0.21528525 | 0.04110771 |
| GO:0032464~positive regulation of protein homooligomerization | 4 | 0.21528525 | 0.04110771 |
| GO:0006307~DNA dealkylation involved in DNA repair | 4 | 0.21528525 | 0.04110771 |
| GO:0070102~interleukin-6-mediated signaling pathway | 4 | 0.21528525 | 0.04110771 |
| GO:0031110~regulation of microtubule polymerization or depolymerization | 4 | 0.21528525 | 0.04110771 |
| GO:0007067~mitotic nuclear division | 32 | 1.72228202 | 0.04123376 |
| GO:0009636~response to toxic substance | 14 | 0.75349839 | 0.04181234 |
| GO:0045669~positive regulation of osteoblast differentiation | 11 | 0.59203445 | 0.04193419 |
| GO:0097421~liver regeneration | 7 | 0.37674919 | 0.04247983 |
| GO:0071549~cellular response to dexamethasone stimulus | 7 | 0.37674919 | 0.04247983 |
| GO:0045736~negative regulation of cyclin-dependent protein serine/threonine kinase activity | 6 | 0.32292788 | 0.04337866 |
| GO:0071395~cellular response to jasmonic acid stimulus | 3 | 0.16146394 | 0.04346436 |
| GO:0031508~pericentric heterochromatin assembly | 3 | 0.16146394 | 0.04346436 |
| GO:0071866~negative regulation of apoptotic process in bone marrow | 3 | 0.16146394 | 0.04346436 |
| GO:0021861~forebrain radial glial cell differentiation | 3 | 0.16146394 | 0.04346436 |
| GO:0032275~luteinizing hormone secretion | 3 | 0.16146394 | 0.04346436 |
| GO:0051835~positive regulation of synapse structural plasticity | 3 | 0.16146394 | 0.04346436 |
| GO:1900746~regulation of vascular endothelial growth factor signaling pathway | 3 | 0.16146394 | 0.04346436 |
| GO:2001269~positive regulation of cysteine-type endopeptidase activity involved in apoptotic signaling pathway | 3 | 0.16146394 | 0.04346436 |
| GO:0043161~proteasome-mediated ubiquitin-dependent protein catabolic process | 27 | 1.45317546 | 0.04512701 |
| GO:0001822~kidney development | 14 | 0.75349839 | 0.04534291 |
| GO:0048813~dendrite morphogenesis | 8 | 0.43057051 | 0.04567874 |
| GO:0071363~cellular response to growth factor stimulus | 9 | 0.48439182 | 0.04693712 |
| GO:0016569~covalent chromatin modification | 17 | 0.91496233 | 0.04821708 |
| GO:0033138~positive regulation of peptidyl-serine phosphorylation | 12 | 0.64585576 | 0.04924341 |
| GO:0008284~positive regulation of cell proliferation | 54 | 2.90635091 | 0.04945363 |
