# Supplementary material for: Analysis of METTL3 and METTL14 in hepatocellular carcinoma
Source: Aging (Albany NY). 2020 Nov 6;12(21):21638–59. doi: 10.18632/aging.103959 (PMC7695415; doi:10.18632/aging.103959)
Supplement: Supplementary Table 8 [file aging-12-103959-s005..docx]

**Supplementary Table 8. GO analysis of TEGs regulated by METTL14.**

| Term | Count | % | PValue |
| --- | --- | --- | --- |
| GO:0007186~G-protein coupled receptor signaling pathway | 203 | 8.63829787 | 6.56E-22 |
| GO:0050911~detection of chemical stimulus involved in sensory perception of smell | 117 | 4.9787234 | 1.27E-19 |
| GO:0007608~sensory perception of smell | 44 | 1.87234043 | 6.19E-08 |
| GO:0007268~chemical synaptic transmission | 53 | 2.25531915 | 5.20E-06 |
| GO:0008380~RNA splicing | 39 | 1.65957447 | 2.59E-05 |
| GO:0007267~cell-cell signaling | 51 | 2.17021277 | 1.10E-04 |
| GO:0050907~detection of chemical stimulus involved in sensory perception | 24 | 1.0212766 | 4.14E-04 |
| GO:0071345~cellular response to cytokine stimulus | 10 | 0.42553191 | 4.43E-04 |
| GO:0008045~motor neuron axon guidance | 9 | 0.38297872 | 7.37E-04 |
| GO:0042742~defense response to bacterium | 31 | 1.31914894 | 0.00106885 |
| GO:0008284~positive regulation of cell proliferation | 77 | 3.27659574 | 0.00123807 |
| GO:0071435~potassium ion export | 6 | 0.25531915 | 0.00173926 |
| GO:0030198~extracellular matrix organization | 38 | 1.61702128 | 0.00178814 |
| GO:0042310~vasoconstriction | 8 | 0.34042553 | 0.00186327 |
| GO:0019221~cytokine-mediated signaling pathway | 28 | 1.19148936 | 0.00196031 |
| GO:0006357~regulation of transcription from RNA polymerase II promoter | 72 | 3.06382979 | 0.00241312 |
| GO:0009952~anterior/posterior pattern specification | 19 | 0.80851064 | 0.00397716 |
| GO:0043010~camera-type eye development | 12 | 0.5106383 | 0.00461495 |
| GO:0007218~neuropeptide signaling pathway | 22 | 0.93617021 | 0.00528883 |
| GO:0007193~adenylate cyclase-inhibiting G-protein coupled receptor signaling pathway | 13 | 0.55319149 | 0.0060609 |
| GO:0008016~regulation of heart contraction | 10 | 0.42553191 | 0.00685774 |
| GO:0030534~adult behavior | 9 | 0.38297872 | 0.00726989 |
| GO:0045892~negative regulation of transcription, DNA-templated | 77 | 3.27659574 | 0.00730373 |
| GO:0007409~axonogenesis | 21 | 0.89361702 | 0.00790726 |
| GO:0031581~hemidesmosome assembly | 6 | 0.25531915 | 0.00811136 |
| GO:0009653~anatomical structure morphogenesis | 20 | 0.85106383 | 0.00833186 |
| GO:0031665~negative regulation of lipopolysaccharide-mediated signaling pathway | 5 | 0.21276596 | 0.00851334 |
| GO:0000398~mRNA splicing, via spliceosome | 39 | 1.65957447 | 0.00861721 |
| GO:0048535~lymph node development | 7 | 0.29787234 | 0.00951746 |
| GO:0006171~cAMP biosynthetic process | 7 | 0.29787234 | 0.00951746 |
| GO:0007605~sensory perception of sound | 26 | 1.10638298 | 0.00961727 |
| GO:0045893~positive regulation of transcription, DNA-templated | 78 | 3.31914894 | 0.01081035 |
| GO:0061337~cardiac conduction | 12 | 0.5106383 | 0.01181569 |
| GO:0007192~adenylate cyclase-activating serotonin receptor signaling pathway | 6 | 0.25531915 | 0.01194116 |
| GO:0007210~serotonin receptor signaling pathway | 6 | 0.25531915 | 0.01194116 |
| GO:0042711~maternal behavior | 6 | 0.25531915 | 0.01194116 |
| GO:0006935~chemotaxis | 24 | 1.0212766 | 0.01220986 |
| GO:0086011~membrane repolarization during action potential | 4 | 0.17021277 | 0.01291332 |
| GO:0045741~positive regulation of epidermal growth factor-activated receptor activity | 5 | 0.21276596 | 0.0139326 |
| GO:0007187~G-protein coupled receptor signaling pathway, coupled to cyclic nucleotide second messenger | 12 | 0.5106383 | 0.0139476 |
| GO:0007626~locomotory behavior | 18 | 0.76595745 | 0.01478496 |
| GO:0034765~regulation of ion transmembrane transport | 22 | 0.93617021 | 0.01552762 |
| GO:0050918~positive chemotaxis | 10 | 0.42553191 | 0.0157332 |
| GO:0007165~signal transduction | 159 | 6.76595745 | 0.01580292 |
| GO:0035235~ionotropic glutamate receptor signaling pathway | 8 | 0.34042553 | 0.01606379 |
| GO:0002323~natural killer cell activation involved in immune response | 7 | 0.29787234 | 0.01704855 |
| GO:0033141~positive regulation of peptidyl-serine phosphorylation of STAT protein | 7 | 0.29787234 | 0.01704855 |
| GO:0000122~negative regulation of transcription from RNA polymerase II promoter | 103 | 4.38297872 | 0.01704918 |
| GO:0007010~cytoskeleton organization | 29 | 1.23404255 | 0.017887 |
| GO:0035176~social behavior | 12 | 0.5106383 | 0.01906536 |
| GO:0045087~innate immune response | 65 | 2.76595745 | 0.02053961 |
| GO:0071805~potassium ion transmembrane transport | 23 | 0.9787234 | 0.02082913 |
| GO:0086013~membrane repolarization during cardiac muscle cell action potential | 5 | 0.21276596 | 0.02112234 |
| GO:0043268~positive regulation of potassium ion transport | 5 | 0.21276596 | 0.02112234 |
| GO:0045616~regulation of keratinocyte differentiation | 5 | 0.21276596 | 0.02112234 |
| GO:0006405~RNA export from nucleus | 13 | 0.55319149 | 0.02141475 |
| GO:0042633~hair cycle | 6 | 0.25531915 | 0.02289311 |
| GO:1901379~regulation of potassium ion transmembrane transport | 6 | 0.25531915 | 0.02289311 |
| GO:1901381~positive regulation of potassium ion transmembrane transport | 6 | 0.25531915 | 0.02289311 |
| GO:0006397~mRNA processing | 31 | 1.31914894 | 0.02356585 |
| GO:0032229~negative regulation of synaptic transmission, GABAergic | 4 | 0.17021277 | 0.02361679 |
| GO:0007601~visual perception | 34 | 1.44680851 | 0.02407 |
| GO:0045944~positive regulation of transcription from RNA polymerase II promoter | 134 | 5.70212766 | 0.0275816 |
| GO:0030520~intracellular estrogen receptor signaling pathway | 7 | 0.29787234 | 0.02789476 |
| GO:0018108~peptidyl-tyrosine phosphorylation | 27 | 1.14893617 | 0.02850089 |
| GO:0030574~collagen catabolic process | 14 | 0.59574468 | 0.02974786 |
| GO:0006275~regulation of DNA replication | 6 | 0.25531915 | 0.03020089 |
| GO:0044130~negative regulation of growth of symbiont in host | 6 | 0.25531915 | 0.03020089 |
| GO:0007340~acrosome reaction | 6 | 0.25531915 | 0.03020089 |
| GO:0046488~phosphatidylinositol metabolic process | 5 | 0.21276596 | 0.03020666 |
| GO:0050832~defense response to fungus | 8 | 0.34042553 | 0.03032825 |
| GO:0007417~central nervous system development | 22 | 0.93617021 | 0.03442216 |
| GO:0031334~positive regulation of protein complex assembly | 7 | 0.29787234 | 0.0347086 |
| GO:0048666~neuron development | 11 | 0.46808511 | 0.03482443 |
| GO:0001580~detection of chemical stimulus involved in sensory perception of bitter taste | 10 | 0.42553191 | 0.03607057 |
| GO:0007155~cell adhesion | 67 | 2.85106383 | 0.03640495 |
| GO:0007198~adenylate cyclase-inhibiting serotonin receptor signaling pathway | 3 | 0.12765957 | 0.03705384 |
| GO:0033563~dorsal/ventral axon guidance | 3 | 0.12765957 | 0.03705384 |
| GO:0007200~phospholipase C-activating G-protein coupled receptor signaling pathway | 14 | 0.59574468 | 0.03733063 |
| GO:0006936~muscle contraction | 20 | 0.85106383 | 0.03746527 |
| GO:0009913~epidermal cell differentiation | 4 | 0.17021277 | 0.03781647 |
| GO:1903140~regulation of establishment of endothelial barrier | 4 | 0.17021277 | 0.03781647 |
| GO:0072205~metanephric collecting duct development | 4 | 0.17021277 | 0.03781647 |
| GO:0060307~regulation of ventricular cardiac muscle cell membrane repolarization | 6 | 0.25531915 | 0.03881899 |
| GO:0006376~mRNA splice site selection | 6 | 0.25531915 | 0.03881899 |
| GO:1903507~negative regulation of nucleic acid-templated transcription | 11 | 0.46808511 | 0.03981552 |
| GO:0051781~positive regulation of cell division | 11 | 0.46808511 | 0.03981552 |
| GO:0043517~positive regulation of DNA damage response, signal transduction by p53 class mediator | 5 | 0.21276596 | 0.04125432 |
| GO:0060306~regulation of membrane repolarization | 5 | 0.21276596 | 0.04125432 |
| GO:0021702~cerebellar Purkinje cell differentiation | 5 | 0.21276596 | 0.04125432 |
| GO:0042445~hormone metabolic process | 5 | 0.21276596 | 0.04125432 |
| GO:0060707~trophoblast giant cell differentiation | 5 | 0.21276596 | 0.04125432 |
| GO:0002687~positive regulation of leukocyte migration | 5 | 0.21276596 | 0.04125432 |
| GO:0048024~regulation of mRNA splicing, via spliceosome | 5 | 0.21276596 | 0.04125432 |
| GO:0045662~negative regulation of myoblast differentiation | 7 | 0.29787234 | 0.04250801 |
| GO:0007411~axon guidance | 27 | 1.14893617 | 0.04326177 |
| GO:0006351~transcription, DNA-templated | 250 | 10.6382979 | 0.04637018 |
| GO:0007275~multicellular organism development | 74 | 3.14893617 | 0.04759029 |
| GO:0007141~male meiosis I | 6 | 0.25531915 | 0.04878682 |
