# Supplementary material for: Analysis of METTL3 and METTL14 in hepatocellular carcinoma
Source: Aging (Albany NY). 2020 Nov 6;12(21):21638–59. doi: 10.18632/aging.103959 (PMC7695415; doi:10.18632/aging.103959)
Supplement: Supplementary Tables 9, 10, 11 and 12 [file aging-12-103959-s006..pdf]

# SUPPLEMENTARY TABLES

**Supplementary Table 9. The correlations between the expression of hub genes and METTL3 expression in HCC.**

| Gene | R | P value | Gene | R | P value |
|---|---|---|---|---|---|
| MYBL2 | 0.41 | <0.001 | KIF18B | 0.54 | <0.001 |
| FOXM1 | 0.51 | <0.001 | TACC3 | 0.41 | <0.001 |
| TRIP12 | 0.53 | <0.001 | CCNB2 | 0.5 | <0.001 |
| HECTD1 | 0.52 | <0.001 | CDT1 | 0.43 | <0.001 |
| SKP1 | 0.31 | <0.001 | SPC25 | 0.45 | <0.001 |
| FBXW9 | 0.33 | <0.001 | CDC25C | 0.4 | <0.001 |
| CBLB | 0.43 | <0.001 | ASF1B | 0.4 | <0.001 |
| FBXL16 | 0.062 | 0.24 | UBE3A | 0.4 | <0.001 |
| KLHL25 | 0.24 | <0.001 | FBXL14 | 0.41 | <0.001 |
| ESPL1 | 0.48 | <0.001 | ASB8 | 0.48 | <0.001 |
| KLHL13 | 0.22 | <0.001 | DTX3L | 0.4 | <0.001 |
| CDCA5 | 0.53 | <0.001 | FZR1 | 0.39 | <0.001 |
| SIAH2 | 0.18 | <0.001 | MGRN1 | 0.34 | <0.001 |
| FBXL15 | 0.1 | 0.047 | TRAIP | 0.5 | <0.001 |
| KIFC1 | 0.55 | <0.001 | TROAP | 0.46 | <0.001 |

**Supplementary Table 10. The associations between the expression of hub genes and overall survival of HCC patients.**

| Gene | HR (95%CI) | P value | Gene | HR (95%CI) | P value |
|---|---|---|---|---|---|
| MYBL2 | 2.29 (1.62-3.24) | <0.001 | KIF18B | 2.13 (1.49-3.03) | <0.001 |
| FOXM1 | 1.91 (1.33-2.74) | <0.001 | TACC3 | 1.8 (1.27-2.55) | <0.001 |
| TRIP12 | 1.27 (0.86-1.88) | 0.23 | CCNB2 | 1.91 (1.28-2.87) | 0.0013 |
| HECTD1 | 0.63 (0.44-0.89) | 0.008 | CDT1 | 2.05 (1.45-2.9) | <0.001 |
| SKP1 | 0.75 (0.53-1.08) | 0.12 | SPC25 | 2.13 (1.51-3.02) | <0.001 |
| FBXW9 | 1.43 (0.95-2.14) | 0.084 | CDC25C | 1.92 (1.36-2.71) | <0.001 |
| CBLB | 1.2 (0.85-1.69) | 0.3 | ASF1B | 1.71 (1.21-2.42) | 0.002 |
| FBXL16 | 0.77 (0.53-1.12) | 0.17 | UBE3A | 0.54 (0.38-0.78) | <0.001 |
| KLHL25 | 1.29 (0.89-1.88) | 0.17 | FBXL14 | 0.59 (0.41-0.84) | 0.0033 |
| ESPL1 | 1.92 (1.36-2.72) | <0.001 | ASB8 | 0.75 (0.53-1.06) | 0.099 |
| KLHL13 | 1.18 (0.83-1.69) | 0.36 | DTX3L | 0.62 (0.41-0.93) | 0.021 |
| CDCA5 | 2.32 (1.62-3.32) | <0.001 | FZR1 | 0.72 (0.48-1.07) | 0.1 |
| SIAH2 | 0.54 (0.38-0.78) | <0.001 | MGRN1 | 0.54 (0.35-0.82) | 0.0035 |
| FBXL15 | 0.61 (0.42-0.89) | 0.0091 | TRAIP | 1.98 (1.38-2.85) | <0.001 |
| KIFC1 | 2.08 (1.47-2.93) | <0.001 | TROAP | 1.84 (1.27-2.66) | 0.001 |

**Supplementary Table 11. The correlations between the expression of hub genes and METTL14 expression in HCC.**

| Gene | R | P value | Gene | R | P value |
|---|---|---|---|---|---|
| HNRNPA2B1 | 0.52 | <0.001 | RBM17 | 0.23 | <0.001 |
| HSPA8 | 0.38 | <0.001 | PPIH | 0.11 | 0.036 |
| MPHOSPH10 | 0.42 | <0.001 | BUB1 | 0.35 | <0.001 |
| DDX55 | 0.43 | <0.001 | GPATCH4 | 0.3 | <0.001 |
| SRRM1 | 0.7 | <0.001 | NOP56 | -0.077 | 0.14 |
| SF1 | 0.73 | <0.001 | SNRNP27 | 0.58 | <0.001 |
| UTP14A | 0.35 | <0.001 | BCCIP | 0.16 | 0.017 |
| HNRNPR | 0.52 | <0.001 | WDR75 | 0.35 | <0.001 |
| EXOSC5 | -0.11 | 0.027 | NCL | 0.46 | <0.001 |
| SF3A2 | 0.11 | 0.036 | DHX40 | 0.54 | <0.001 |
| DDX31 | 0.41 | <0.001 | WDR43 | 0.51 | <0.001 |
| WDR74 | -0.094 | 0.07 | TRUB1 | 0.62 | <0.001 |
| DDX47 | 0.45 | <0.001 | SSB | 0.25 | <0.001 |
| CEBPZ | 0.17 | <0.001 | SDAD1 | 0.75 | <0.001 |
| UTP3 | 0.56 | <0.001 | PWP2 | 0.17 | 0.0014 |

**Supplementary Table 12. The associations between the expression of hub genes and overall survival of HCC patients.**

| Gene | HR (95%CI) | P value | Gene | HR (95%CI) | P value |
|---|---|---|---|---|---|
| HNRNPA2B1 | 1.38 (0.95-2.01) | 0.094 | RBM17 | 1.86 (1.32-2.63) | <0.001 |
| HSPA8 | 1.81 (1.21-2.71) | 0.0036 | PPIH | 1.76 (1.24-2.49) | 0.0012 |
| MPHOSPH10 | 0.78 (0.53-1.13) | 0.19 | BUB1 | 2.1 (1.45-3.04) | <0.001 |
| DDX55 | 1.8 (1.26-2.56) | 0.001 | GPATCH4 | 1.55 (1.09-2.18) | 0.013 |
| SRRM1 | 1.27 (0.9-1.79) | 0.18 | NOP56 | 2.33 (1.65-3.3) | <0.001 |
| SF1 | 0.82 (0.58-1.16) | 0.26 | SNRNP27 | 1.28 (0.9-1.83) | 0.17 |
| UTP14A | 1.46 (1.03-2.06) | 0.03 | BCCIP | 1.55 (1.1-2.19) | 0.012 |
| HNRNPR | 1.81 (1.27-2.6) | <0.001 | WDR75 | 2.09 (1.45-3.02) | <0.001 |
| EXOSC5 | 1.33 (0.91-1.95) | 0.14 | NCL | 1.7 (1.19-2.44) | 0.0031 |
| SF3A2 | 1.43 (1-2.05) | 0.048 | DHX40 | 0.68 (0.47-0.98) | 0.036 |
| DDX31 | 1.76 (1.21-2.54) | 0.0024 | WDR43 | 1.64 (1.15-2.33) | 0.0055 |
| WDR74 | 0.81 (0.57-1.16) | 0.26 | TRUB1 | 0.76 (0.54-1.08) | 0.13 |
| DDX47 | 1.27 (0.9-1.8) | 0.18 | SSB | 1.95 (1.35-2.83) | <0.001 |
| CEBPZ | 1.51 (1.06-2.15) | 0.022 | SDAD1 | 1.39 (0.97-2.01) | 0.072 |
| UTP3 | 0.8 (0.57-1.14) | 0.22 | PWP2 | 1.34 (0.95-1.89) | 0.094 |
